# Caspase-4 is essential for saikosaponin a-induced apoptosis acting upstream of caspase-2 and γ-H2AX in colon cancer cells

**DOI:** 10.18632/oncotarget.22247

**Published:** 2017-11-01

**Authors:** Su Jin Kang, Young Joon Lee, Sung Gu Kang, Soyoung Cho, Wonsuck Yoon, Ji Hong Lim, Sang-Hyun Min, Tae Ho Lee, Byeong Mo Kim

**Affiliations:** ^1^ The Medical Research Center for Globalization of Herbal Medicine, Daegu Haany University, Gyeongsan, Gyeongsangbuk-Do 38610, Republic of Korea; ^2^ Department of Preventive Medicine, College of Korean Medicine, Daegu Haany University, Gyeongsan, Gyeongsangbuk-Do 38610, Republic of Korea; ^3^ Department of Urology, Korea University College of Medicine, Seongbuk-gu, Seoul 02841, Republic of Korea; ^4^ Department of Science for Aging, Yonsei University, Seodaemun-gu, Seoul 03722, Republic of Korea; ^5^ Severance Integrative Research Institute for Cerebral & Cardiovascular Diseases (SIRIC), Yonsei University College of Medicine, Seodaemun-gu, Seoul 03722, Republic of Korea; ^6^ Allergy Immunology Center, Korea University College of Medicine, Seongbuk-gu, Seoul 02841, Republic of Korea; ^7^ Department of Biomedical Chemistry, Konkuk University, Chungju, Chungbuk 27478, Republic of Korea; ^8^ New Drug Development Center, DGMIF, Dong-gu, Daegu 41061, Republic of Korea; ^9^ Division of Gerontology, Department of Medicine, Beth Israel Deaconess Medical Center, Harvard Medical School, Boston, MA 02215, USA

**Keywords:** saikosaponin a (SSa), human colon carcinoma (HCC), endoplasmic reticulum (ER) stress, caspase-4, DNA damage

## Abstract

Saikosaponin a (SSa), a bioactive phytochemical from *Bupleurum*, triggers sequential caspase-2 and caspase-8 activation, and thereby induces caspase-mediated apoptosis in human colon carcinoma (HCC) cells. However, the upstream mechanism of caspase-2 activation remains unknown. Therefore, we investigated the signaling mechanisms underlying SSa-induced caspase activation and apoptosis in HCC cells. SSa treatment triggered marked antitumor effects, especially in HCC cells, in a cell culture model and a mouse xenograft model. SSa also induced the activation of several endoplasmic reticulum (ER) stress signals. Specifically, caspase-4, a critical regulator of ER stress-induced apoptosis, was activated significantly after SSa treatment. Mechanistically, selective inhibition of caspase-4 suppressed SSa-induced apoptosis, colony inhibition, and the activation of caspase-3, -8, and -2, but not *vice versa*. Consistent with the important role of caspase-2 in the DNA damage response, SSa induced DNA damage, as evidenced by a cytokinesis-block micronucleus assay, single-cell gel electrophoresis, and an increase in the levels of γ-H2AX, a DNA damage marker. Moreover, inhibition of caspase-4 activation inhibited SSa-induced histone H2AX phosphorylation. Taken together, these results suggest that caspase-4 is an upstream regulator of SSa-induced DNA damage and caspase activation in HCC cells. Given that SSa-induced apoptosis appeared to be specific to certain cell types including HCC cells, SSa may be a promising cancer therapy agent in certain types of cancer.

## INTRODUCTION

Human caspase-4 and mouse caspase-12, which are localized in the endoplasmic reticulum (ER), are cleaved by ER stress-inducing agents and are involved in the ER stress response [1-3]. Although the function of caspase-4 is not fully understood, it is believed to play a critical role in the activation of inflammasomes [4], which are components of the immune system. Caspase-4 is also involved in Fas- and TRAIL-induced apoptosis [5, 6]. Moreover, caspase-4 activation is essential for ER stress-induced apoptosis in some cell lines [1, 7-9]. However, at least one study has suggested that caspase-4 is not essential for ER stress-induced apoptosis [10]; therefore, the role of caspase-4 in ER stress-induced apoptosis remains unclear.

Saikosaponin a (SSa), a triterpenoid glycoside from *Bupleurum*, exhibits various biological activities, such as anti-inflammatory, analgesic, neuromodulatory, antifibrotic, antiepileptic, and anti-cancer activity. SSa can inhibit cell growth and DNA synthesis, and induce apoptosis [11-13]. Previously, our group found that SSa could induce the activation of various caspases and poly (ADP-ribose) polymerase (PARP), decrease Bcl-2 and X-linked inhibitor of apoptosis (XIAP) expression, and induce apoptosis in human colon carcinoma (HCC) cells [14]. In addition, SSa has been shown to have cytotoxicity against human gastric cancer, breast cancer, and hepatoma [15-18].

Caspase-2, one of the most evolutionarily conserved caspases, has multiple functions in response to DNA damage [19, 20]. Caspase-2 deficiency leads to genetic instability and triggers an aberrant DNA damage response [21]. Specifically, some studies have shown that caspase-2 is necessary for DNA damage-induced apoptosis [19-23]. Previously, we showed that sequential caspase-2 and -8 activation was important for SSa-induced apoptosis in HCC cells [14]. Although caspase-2 is generally classified as an initiator caspase, its cleavage specificity is closer to the effector caspases, such as caspase-3 and -7 [24].

In our study, we found that SSa triggered apoptotic cell death in a cell type-specific manner and was dependent on caspase-4 activation. Our results also show that caspase-4 is an upstream regulator of DNA damage response and/or DNA damage-mediated sequential caspase-2 and -8 activation.

## RESULTS

### SSa triggers cell type-dependent cell death

**Figure 1:**
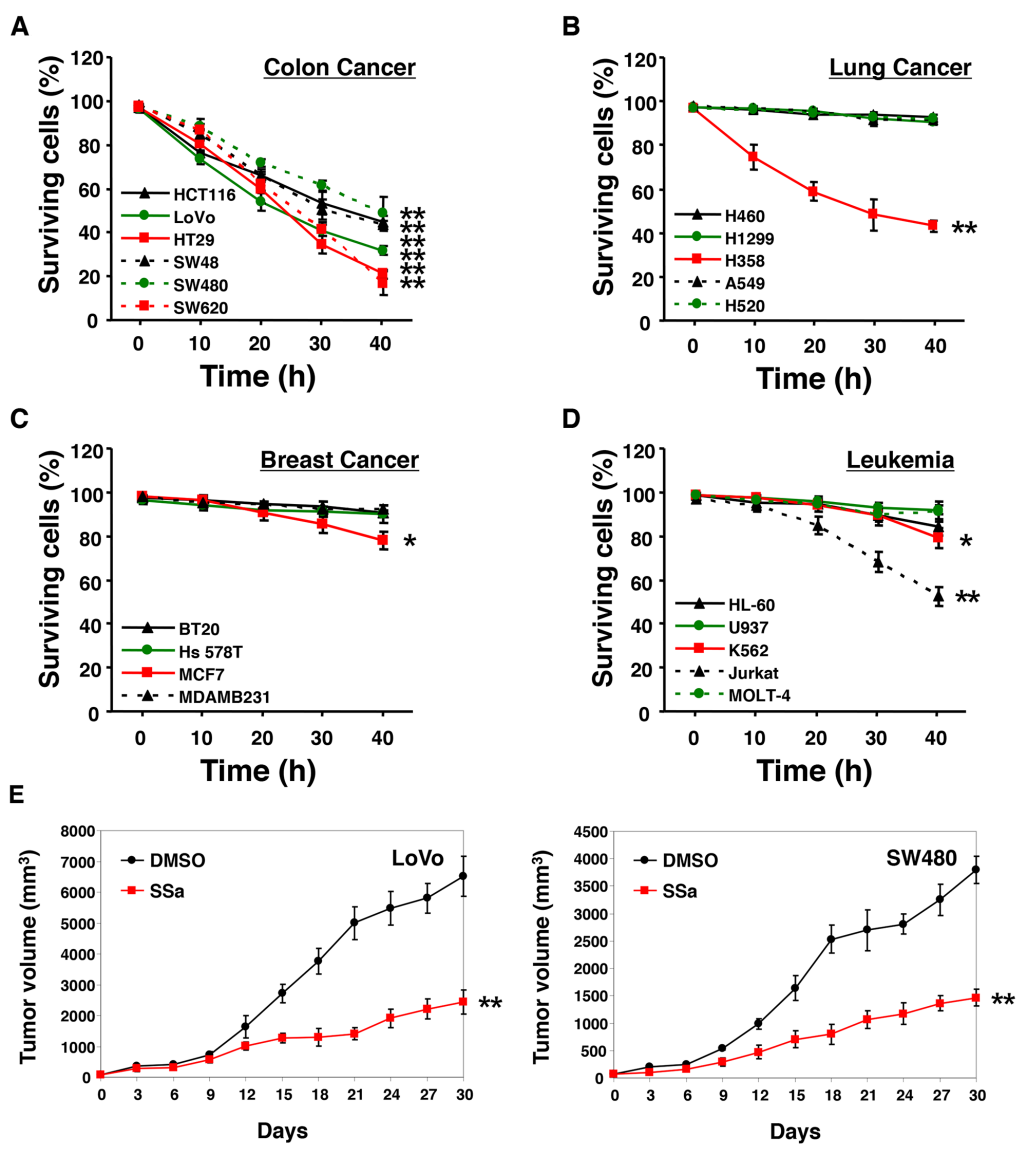
Effect of SSa on cell viability Human colon cancer **(A)**, lung cancer **(B)**, breast cancer **(C)**, and leukemia **(D)** cell lines were treated with 20 μM SSa for up to 40 h. Cell viability was assessed using Trypan Blue. Values on the y-axis are surviving cells as a percentage of the total population after SSa treatment. Results are the mean ± standard error from three experiments. ^*^P < 0.05 and ^**^P < 0.01 compared with the 0-h control. The Mann-Whitney *U* test was used for statistical analysis. **(E)** Nude mice (6 weeks old) with subcutaneous LoVo or SW480 colon carcinoma xenografts (65–80 mm^3^) were pretreated with a vehicle (10% DMSO in PBS, pH 7.4) or SSa (2 mg/kg) once weekly beginning on day 0. Data shown represent the mean ± standard errors of two independent experiments, each using five mice per group.

According to previous studies [15-17], SSa has cytotoxic effects against some breast carcinoma and hepatoma carcinoma cells. We also reported SSa cytotoxicity in HCC cells [14]. To investigate whether SSa has universal cytotoxicity in cancer cells, various cancer cells from different tissue types were treated with SSa. As shown in Figure 1, when exposed to SSa, all colon cancer cell populations showed significantly decreased cell viability, whereas lung cancer cell populations except H358, breast cancer cell populations except MCF-7, and leukemia cell populations except Jurkat and K562 exhibited minimally decreased cell viability. The same results were obtained when cell lines were assayed for apoptosis using sub-G1 fraction analysis and Hoechst 33342 staining (data not shown). These results indicate that SSa can trigger apoptotic cancer cell death in a cell type-dependent manner. Since SSa reduced cell viability in all HCC cell lines, SSa may be effective for the treatment of colon cancer. Additionally, SSa concentrations of less than 10 μM had little to no effect on the cell viability of all colon cancer cell lines tested (Supplementary Figure 1). At 10 μM and 15 μM, SSa caused mild and moderate cytotoxicity, respectively. Because 20 μM SSa led to significant cytotoxicity in all colon cancer cells tested, we used this concentration for subsequent experiments. Thus, there were both dose-dependent and time-dependent decreases in cell viability in the presence of SSa.

### Prolonged inhibition of tumor growth in a colon carcinoma xenograft model with SSa treatment

To evaluate the *in vivo* antitumor effects of SSa in xenograft models, BALB/c nude mice bearing LoVo or SW480 colon carcinomas on their rear flanks were treated with 2 mg/kg of intraperitoneal SSa once per week. Control mice received injections of the same volume of the vehicle (10% DMSO in PBS, pH 7.4). In mice treated with SSa, prolonged tumor growth inhibition was observed (Figure 1E). The difference between vehicle-treated groups and SSa-treated groups was notable by days 9–12. To verify that SSa treatment had no effect on healthy tissues, we looked for histological changes after the administration of SSa. We found no histopathological signs of damage, such as inflammation of lung, spleen, liver, or kidney tissues after treatment (data not shown).

### SSa activates caspase-4

**Figure 2:**
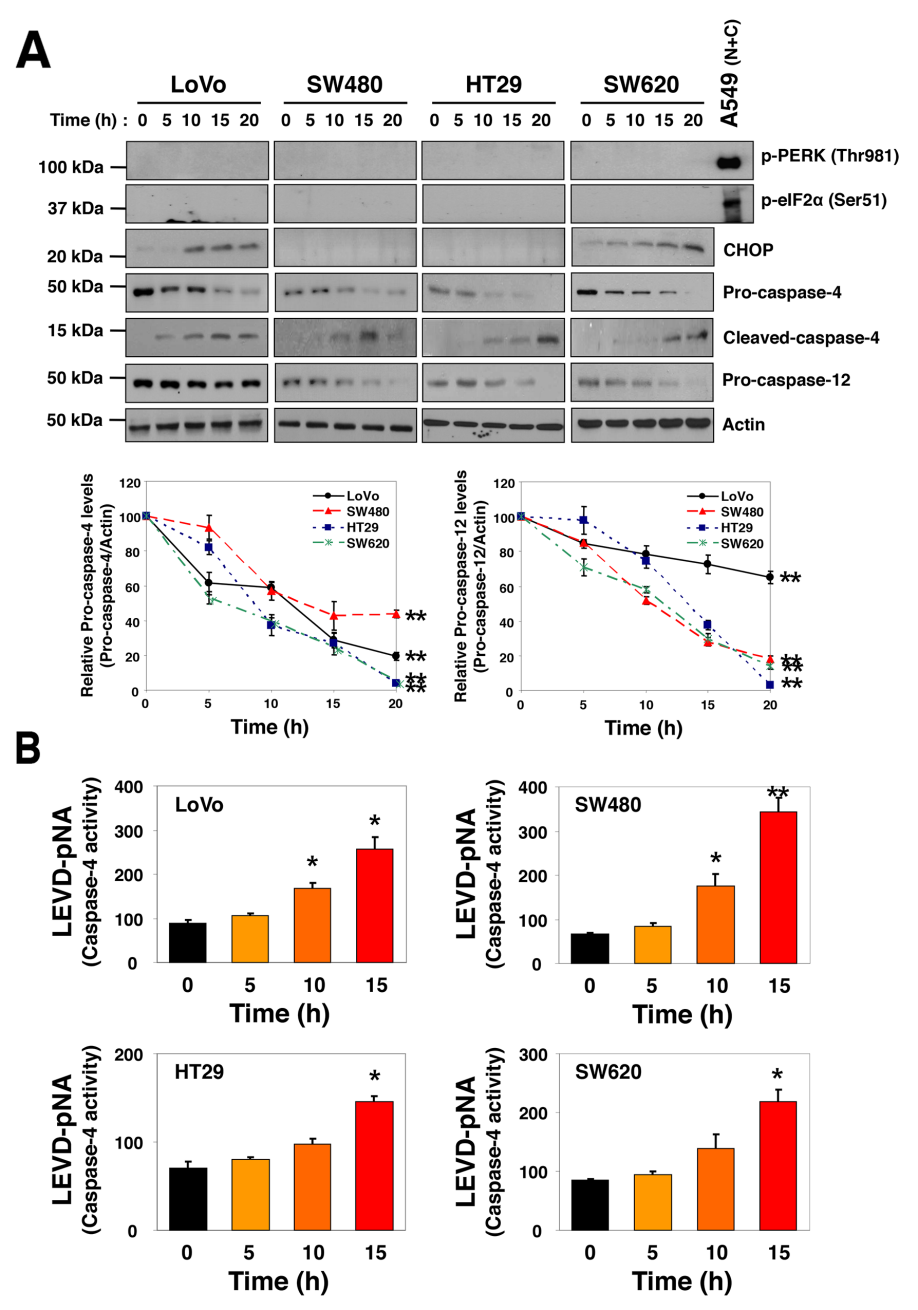
SSa-induced caspase-4 activation in HCC cells LoVo, SW480, HT29, and SW620 cells were treated with 20 μM SSa for the indicated times. **(A)** Levels of phosphorylated PERK (Thr981), phosphorylated eIF2α (Ser51), CHOP, pro-caspase-4, cleaved-caspase-4, and pro-caspase-12 were analyzed by Western blotting. Blots are representative of three independent experiments, and data shown represent the mean ± standard error of three independent experiments. ^**^P < 0.01 compared with the 0-h control. The Mann-Whitney *U* test was used for statistical analysis. A549 lung cancer cell lysate treated with the combination of niflumic acid (100 μM) and ciglitazone 7.5 μM for 30 h was used as a positive control for the phosphorylation of PERK and eIF2α. **(B)** Changes in caspase-4 activity were monitored via detection of liberated pNA from the substrate, LEVD-pNA. All samples were measured in triplicate. Each column represents the mean ± standard error of three independent experiments. ^*^P < 0.05 and ^**^P < 0.01 compared with the 0-h control. The Mann-Whitney *U* test was used for statistical analysis.

SSa and its epimer, saikosaponin d are major triterpenoid saponin derivatives. Because saikosaponin d induces ER stress [25], SSa may also trigger ER stress in HCC cells. To assess this possibility, we investigated several ER-specific signals. SSa increased the expression of ER stress and unfolded protein response genes such as PERK, CHOP, ATF4, and XBP1 in some HCC cells (Supplementary Figure 2). Protein expression assays revealed that levels of CHOP, but not phosphorylated PERK (Thr981) or phosphorylated eIF2α (Ser51), were increased in response to SSa and were cell type-dependent (Figure 2A). For antibody validation, the combination of niflumic acid (100 μM) and ciglitazone 7.5 μM was employed as a positive control, as it has been shown to induce significant phosphorylation of PERK and eIF2α in A549 lung cancer cells in previous research [26]. Interestingly, cleavage of ER-resident caspases, such as caspase-4 and -12, by SSa treatment was significant in all HCC cells (Figure 2A). Moreover, tumor lysates obtained from LoVo and SW480 xenograft mice treated with SSa for 10 days also showed cleavage of caspase-4 (data not shown). Next, we investigated if SSa could increase caspase-4 enzymatic activity. To do this, caspase-4 protease activity was measured using LEVD-pNA, a colorimetric labeled substrate specific for caspase-4. As shown in Figure 2B, SSa activated caspase-4 in a time-dependent manner in human LoVo, SW480, HT29, and SW620 cells.

### Caspase-4 inhibition suppresses SSa-induced activation of caspase-3, caspase-2, and caspase-8, but not vice versa

**Figure 3:**
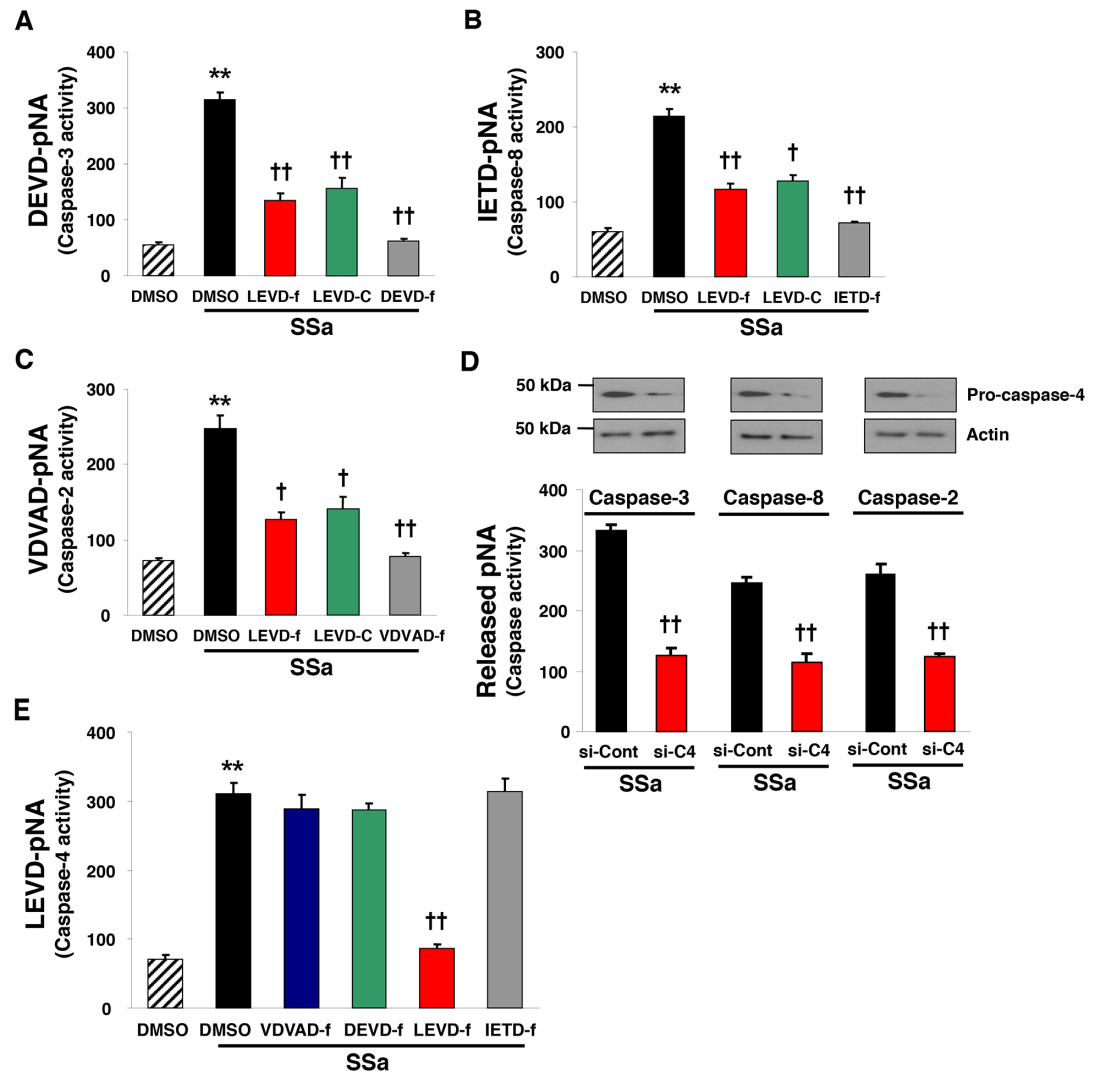
Caspase-4 plays a role in SSa-induced caspase-2, -8, and -3 activation (A–C, E) SW480 cells were treated with 20 μM SSa for 15 h in the presence or absence of caspase-3 inhibitor (z-DEVD-fmk, labeled DEVD-f; 10 μM), caspase-8 inhibitor (z-IETD-fmk, labeled IETD-f; 10 μM), caspase-2 inhibitor (z-VDVAD-fmk, labeled VDVAD-f; 10 μM), or caspase-4 inhibitors (z-LEVD-fmk and Ac-LEVD-CHO, labeled LEVD-f and LEVD-C, respectively; each 10 μM). The activities of caspase-3 **(A)**, caspase-8 **(B)**, caspase-2 **(C)**, and caspase-4 **(E)** were monitored via the detection of pNA liberated from DEVD-pNA, IETD-pNA, VDVAD-pNA, and LEVD-pNA, respectively. Each column represents the mean ± standard error of three independent experiments. ^**^P < 0.01 compared with untreated controls. ^†^*P* < 0.05 and ^††^*P* < 0.01 compared with SSa alone. ANOVA and Tukey’s test were used for statistical analysis. **(D)** SW480 cells were seeded and allowed to reach ∼30% confluence on the day of transfection. Cells were treated with siRNA to caspase-4 or scramble siRNA (each 20 nM) for 30 h, and then exposed to 20 μM SSa for 15 h. Caspase-4 knockdown efficiency was confirmed by Western blotting. Caspase-3, -8, and -2 activities were monitored as in Figure 3A–C. Each column represents the mean ± standard error of three independent experiments. ^††^*P* < 0.01 compared with SSa alone. The Mann-Whitney *U* test was used for statistical analysis.

SSa was shown to induce activation of caspase-3, -8, and -2 in HCC cells in our previous study [14]. Therefore, we examined whether SSa-induced caspase-4 activation was linked with the activation of other caspases. To this end, we used SW480 cells, which we already showed had activated caspase-3, -8, and -2 in the presence of SSa [14]. As shown in Figure 3A-3C, caspase activities (caspase-3, -8, and -2) were enhanced after SSa treatment. However, these effects were attenuated efficiently by treatment with caspase-4 inhibitors (z-LEVD-fmk and Ac-LEVD-CHO). The same results were obtained in LoVo cells (data not shown). Moreover, SSa-induced cleavage of caspase-2 and -8 and Bid truncation (called t-Bid), which is a downstream event of caspase-8 activation, were also inhibited significantly by the caspase-4 inhibitor z-LEVD-fmk in both LoVo and SW480 cells (Supplementary Figure 3). To confirm these results, caspase-4 was silenced using siRNA. Western blot analysis indicated efficient knockdown of caspase-4 (Supplementary Figure 4). Consistent with results in Figure 3A-3C, siRNA against caspase-4 suppressed SSa-induced activation of caspase-3, -8, and -2 (Figure 3D). However, SSa-induced enhancement of caspase-4 activity was not attenuated by z-DEVD-fmk (caspase-3 inhibitor), z-VDVAD-fmk (caspase-2 inhibitor), or z-IETD-fmk (caspase-8 inhibitor) (Figure 3E). Likewise, caspase-3, -2, and -8 knockdown also did not suppress SSa-induced caspase-4 cleavage or enzymatic activation (data not shown). These results suggest that caspase-4 activation occurred upstream of other caspases including caspase-2.

### SSa causes apoptosis in a dose-dependent manner

In our previous study [14], we evaluated SSa-induced apoptosis by analyzing sub-G1 fraction and by Hoechst 33342 staining. Here, we also performed annexin V binding assay to detect redistribution of phosphatidylserine, which is a hallmark for early apoptosis. We treated LoVo and SW480 cells with a range of SSa concentrations. SSa-treated HCC populations contained more annexin V-positive cells as well as more cells with sub-G1 DNA content or condensed/fragmented nuclei compared with the DMSO control (Supplementary Figure 5), indicating that SSa induced dose-dependent apoptosis in HCC cells. Furthermore, SSa caused dose-dependent apoptosis in H358 (the only SSa-sensitive lung cancer cells identified in our experiments) lung cancer cells but not in H1299 (SSa-resistant cells) lung cancer cells, confirming that SSa-induced apoptosis was cell type-specific (Supplementary Figure 5).

### Inhibition of caspase-4 suppresses SSa-induced apoptosis

**Figure 4:**
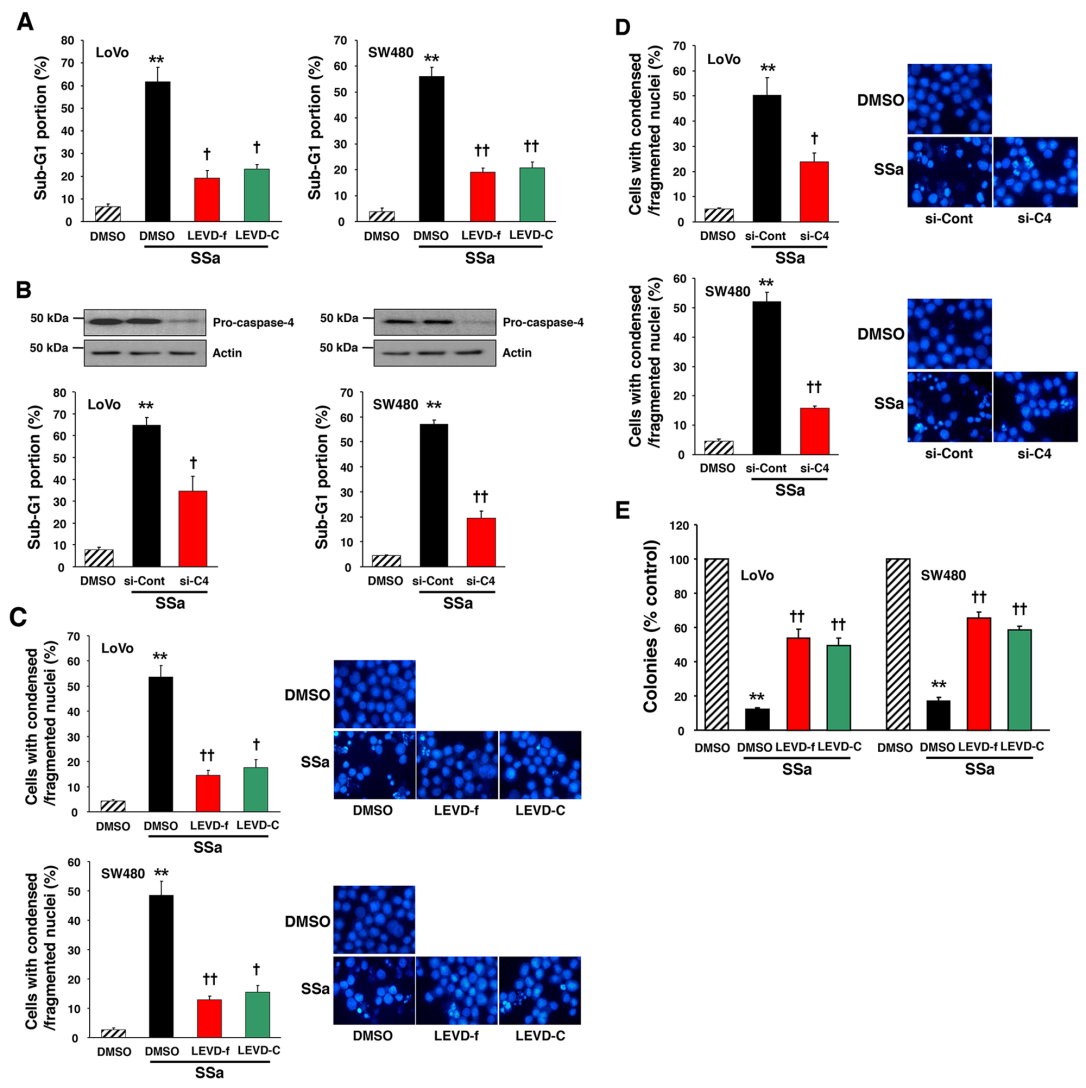
Role of caspase-4 in SSa-induced apoptosis and loss of clonogenic survival **(A and C)** LoVo and SW480 cells were treated with 20 μM SSa for 30 h (for sub-G1 fraction analysis) or 24 h (for Hoechst 33342 staining) in the presence or absence of the caspase-4 inhibitors z-LEVD-fmk (LEVD-f; 10 μM) and Ac-LEVD-CHO (LEVD-C; 10 μM). (A) Cell cycle analysis was used to quantify apoptosis as the percentage of cells in the sub-G1 fraction. Each column represents the mean ± standard error from three experiments. ^**^P < 0.01 compared with untreated controls. ^†^*P* < 0.05 and ^††^*P* < 0.01 compared with SSa alone. ANOVA and Tukey’s test were used for statistical analysis. (C) Morphologically apoptotic cells were quantified after Hoechst 33342 staining. Each column represents the mean ± standard error of cells with condensed/fragmented nuclei in three independent experiments. ^**^P < 0.01 compared with untreated controls. ^†^*P* < 0.05 and ^††^*P* < 0.01 compared with SSa alone. ANOVA and Tukey’s test were used for statistical analysis. Representative images of Hoechst 33342 staining are shown. (B and D) LoVo and SW480 cells were seeded and allowed to reach ∼30% confluence on the day of transfection. Cells were treated with siRNA of caspase-4 (si-C4; 20 nM) or scrambled siRNA (si-Cont; 20 nM) for 30 h and then exposed to 20 μM SSa for 30 h (for sub-G1 fraction analysis) or 24 h (for Hoechst 33342 staining). Caspase-4 knockdown efficiency was confirmed via Western blotting. **(B)** Apoptosis was quantified as in Figure 4A. Each column represents the mean ± standard error from three experiments. ^**^P < 0.01 compared with untreated controls. ^†^*P* < 0.05 and ^††^*P* < 0.01 compared with SSa alone. ANOVA and Tukey’s test were used for statistical analysis. **(D)** Morphologically apoptotic cells were quantified as in Figure 4C. Each column represents the mean ± standard error of three independent experiments. ^**^P < 0.01 compared with untreated controls. ^†^*P* < 0.05 and ^††^*P* < 0.01 compared with SSa alone. ANOVA and Tukey’s test were used for statistical analysis. Representative images of Hoechst 33342 staining are shown. **(E)** LoVo and SW480 cells were treated with 15 μM SSa in the presence or absence of the caspase-4 inhibitors, z-LEVD-fmk (10 μM), and Ac-LEVD-CHO (10 μM). After 12 (for LoVo) or 14 (for SW480) days of incubation, cells were analyzed for cloning efficiency. The number of colonies was expressed as a percentage of the untreated control. Each column represents the mean ± standard error of three independent experiments. ^**^P < 0.01 compared with untreated controls. ^††^*P* < 0.01 compared with SSa alone. ANOVA and Tukey’s test were used for statistical analysis.

Next, to examine the role of caspase-4 in SSa-induced apoptosis, LoVo and SW480 cells were pre-incubated with caspase-4 inhibitors, z-LEVD-fmk or Ac-LEVD-CHO, before exposure to 20 μM SSa. We performed sub-G1 fraction analysis and Hoechst 33342 staining to quantify cells with characteristics of apoptosis. Pre-incubation with z-LEVD-fmk or Ac-LEVD-CHO significantly attenuated SSa-induced increases in the number of apoptotic cells in both LoVo and SW480 cells (Figure 4A and 4C). A significant increase in the percentage of cells in the sub-G1 fraction was observed in SSa-treated LoVo and SW480 cells (61.7 ± 6.5 and 56.2 ± 3.4%, respectively). However, the percentage of cells in the sub-G1 fraction was decreased significantly with z-LEVD-fmk (19.2 ± 3.2 for LoVo; 18.8 ± 1.7% for SW480) and Ac-LEVD-CHO (23.0 ± 2.1 for LoVo; 20.6 ± 2.2% for SW480) treatment. A significant increase in cells with condensed/fragmented nuclei (determined using Hoechst 33342 staining) was also observed in SSa-treated LoVo and SW480 cells (53.7 ± 4.5 and 48.6 ± 4.8%, respectively), whereas the percentage of cells with condensed/fragmented nuclei decreased significantly with z-LEVD-fmk (14.2 ± 2.2 and 12.8 ± 1.3%, respectively) and Ac-LEVD-CHO (17.3 ± 3.1 and 15.3 ± 2.3%, respectively) treatment. Additionally, siRNA against caspase-4 suppressed SSa-induced apoptosis (Figure 4B and 4D), consistent with the results shown in Figure 4A and 4C. A significant increase in the percentage of cells in the sub-G1 fraction was observed in SSa-treated LoVo and SW480 cells (64.8 ± 3.6 and 57.0 ± 1.8%, respectively), whereas the percentage of cells in the sub-G1 fraction decreased significantly with knockdown of caspase-4 (34.4 ± 6.9 and 19.1 ± 2.9%, respectively). A significant increase in cells with condensed and/or fragmented nuclei was also observed when LoVo and SW480 cells were treated with SSa (50.3 ± 7.0 and 52.0 ± 3.2%, respectively). However, these numbers decreased significantly on knockdown of caspase-4 (23.7 ± 3.5 for LoVo; 15.6 ± 0.7% for SW480). These results indicate that caspase-4 activation is intimately linked with the onset of SSa-induced apoptosis. Moreover, given that activation of caspase-2, -8, and -3 is essential for SSa-induced apoptosis [14], these results indicate that the sequential activation of caspase-4, -2, -8, and -3 is a critical step in SSa-induced apoptosis. However, neither the selective caspase-12 inhibitor z-ATAD-fmk (Supplementary Figure 6) nor caspase-12 siRNA (data not shown) attenuated SSa-induced apoptosis.

### Caspase-4 inhibition increases clonogenic survival of colon carcinoma cells in response to SSa treatment

Because caspase-4 inhibitors (z-LEVD-fmk and Ac-LEVD-CHO) inhibited SSa-induced apoptosis, we examined whether these inhibitors could restore clonogenic survival to SSa-treated colon cancer cells. The addition of caspase-4 inhibitors to SSa-treated cells increased clonogenic survival significantly (Figure 4E). Compared with untreated cells, SSa-treated cells had fewer LoVo (12.1 ± 1.1%) and SW480 (17.2 ± 2.3%) colonies, and the addition of z-LEVD-fmk (54.3 ± 5.3% for LoVo; 65.8 ± 3.6% for SW480) or Ac-LEVD-CHO (49.8 ± 4.5% for LoVo; 58.2 ± 2.3% for SW480) to SSa-treated cells increased the number of colonies significantly. These results suggest that the efficiency of clonogenic cell eradication by SSa is dependent on caspase-4 activation. On the other hand, the caspase-12 inhibitor z-ATAD-fmk did not attenuate SSa-triggered eradication of clonogenic cells (data not shown).

### SSa induces micronucleus formation in HCC cells

**Figure 5:**
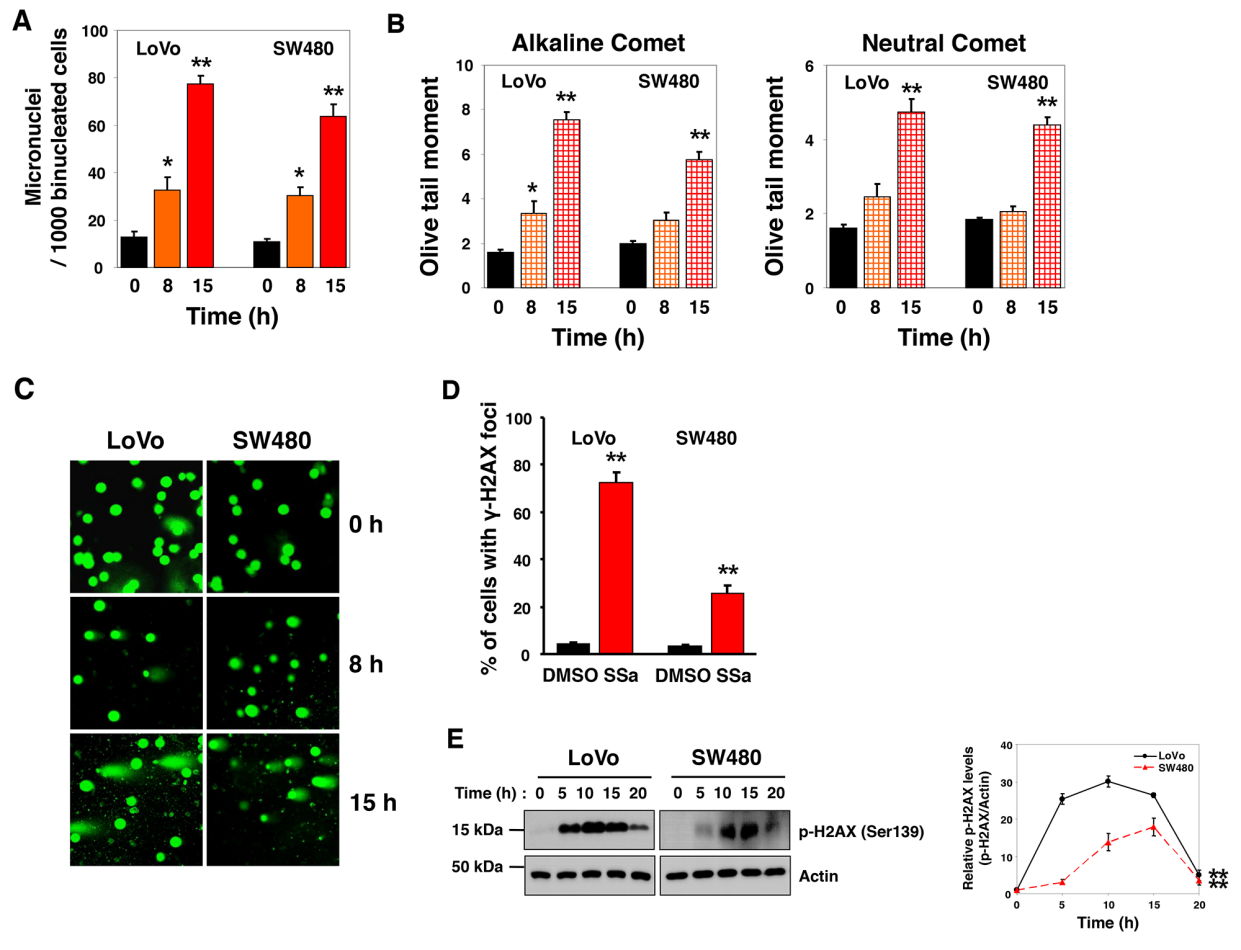
DNA damage induced by SSa treatment of human colon cancer (HCC) cells LoVo and SW480 cells were treated with 20 μM SSa for the indicated times. The micronucleus (MN) test, single-cell gel electrophoresis (SCGE, comet) assay, and ã-H2AX foci assay were used to evaluate genotoxicity associated with SSa exposure. **(A)** MN frequency was determined by analysis of 1000 binucleated cells from each sample. Results are the mean ± standard error from three experiments. ^*^P < 0.05 and ^**^P < 0.01 compared with the 0-h control. The Mann-Whitney *U* test was used for statistical analysis. **(B)** Alkaline and neutral SCGE assays were performed according to standard protocols. A computerized imaging system was used to score and measure DNA comets (OTM). Results are the mean ± standard error from three experiments. ^*^P < 0.05 and ^**^P < 0.01 compared with the 0-h control. The Mann-Whitney *U* test was used for statistical analysis. **(C)** Representative images of SYBR Green staining (1 μg/mL) from the neutral SCGE assay. **(D)** Following SSa treatment for 15 h, anti-phospho-H2AX (Ser139) primary antibody and AlexaFluor 594 secondary antibody were used to immunostain LoVo and SW480 cells. The percentage of cells positive for γ-H2AX foci is shown. Results shown are the mean ± standard error from three experiments. ^**^P < 0.01 compared with the DMSO control. The Mann-Whitney *U* test was used for statistical analysis. **(E)** Western blotting was used to detect H2AX phosphorylation at Ser139. Blots are representative of three independent experiments, and data shown represent the mean ± standard error of three independent experiments. ^**^P < 0.01 compared with the 0-h control. The Mann-Whitney *U* test was used for statistical analysis.

ER stress is associated with DNA damage. Given that SSa activates caspase-2 [14], which has been implicated in the regulation of cell death induced by DNA damage and ER stress [19, 20, 22, 23, 27, 28], we evaluated the effects of SSa on DNA damage and on ER stress. First, formation of micronuclei was used as a biological indicator of SSa-induced genotoxicity. The frequency of micronucleus (MN) formation increased after SSa treatment (Figure 5A and Supplementary Figure 7). After treatment with SSa for 0, 8, and 15 h, average MN frequencies were 13, 33, and 78 per 1000 binucleated cells for LoVo cells, and they were 11, 31, and 64 for SW480 cells, in three independent experiments. These results showed that SSa-induced MN formation was time-dependent.

### SSa induces an increase in the Olive tail moment (OTM) of HCC cells

To further investigate the prevalence of DNA damage, we performed neutral (for DSBs) and alkaline (for SSBs and DSBs) versions of the single-cell gel electrophoresis (SCGE, comet) assay. After treatment with SSa, neutral comet assay results indicated a time-dependent increase in DNA fragmentation in the OTM, indicating the presence of DSBs (Figure 5B, right panel and Figure 5C). Alkaline comet assay results showed that time-dependent increases in SSa-induced SSBs and DSBs in the OTM were more pronounced and were statistically significant (Figure 5B, left panel). The mean percentage of cells with OTM > 5 is shown in Supplementary Figure 8. These data indicate that SSa caused both SSBs and DSBs in HCC cells. The significance of this effect was determined by comparing the DNA quantity and the mean distance of migration in the tail.

### SSa induces the formation of γ-H2AX foci

Phosphorylation of H2AX at Ser139 occurs after DNA damage and is an excellent marker of DNA DSBs [29-31]. Studies have shown that phosphorylated H2AX (called γ-H2AX) is visible as nuclear foci at sites of DNA damage; therefore, we performed immunostaining to ascertain the presence of γ-H2AX foci at sites of DNA DSBs. Using LoVo and SW480 cells, we observed a significantly greater number of γ-H2AX foci-positive cells in SSa-treated cells than in untreated control cells (Figure 5D and Supplementary Figure 9). This was confirmed by Western blotting using anti-phospho-H2AX antibody. Time-dependent H2AX phosphorylation at Ser139 after SSa treatment was observed in these cancer cells (Figure 5E). Moreover, tumor lysates obtained from LoVo and SW480 xenograft mice treated with SSa for 10 days also showed H2AX phosphorylation (data not shown). Collectively, these results show that SSa triggers DNA damage in HCC cells.

### Caspase-4 inhibition suppresses SSa-induced ã-H2AX foci formation

**Figure 6:**
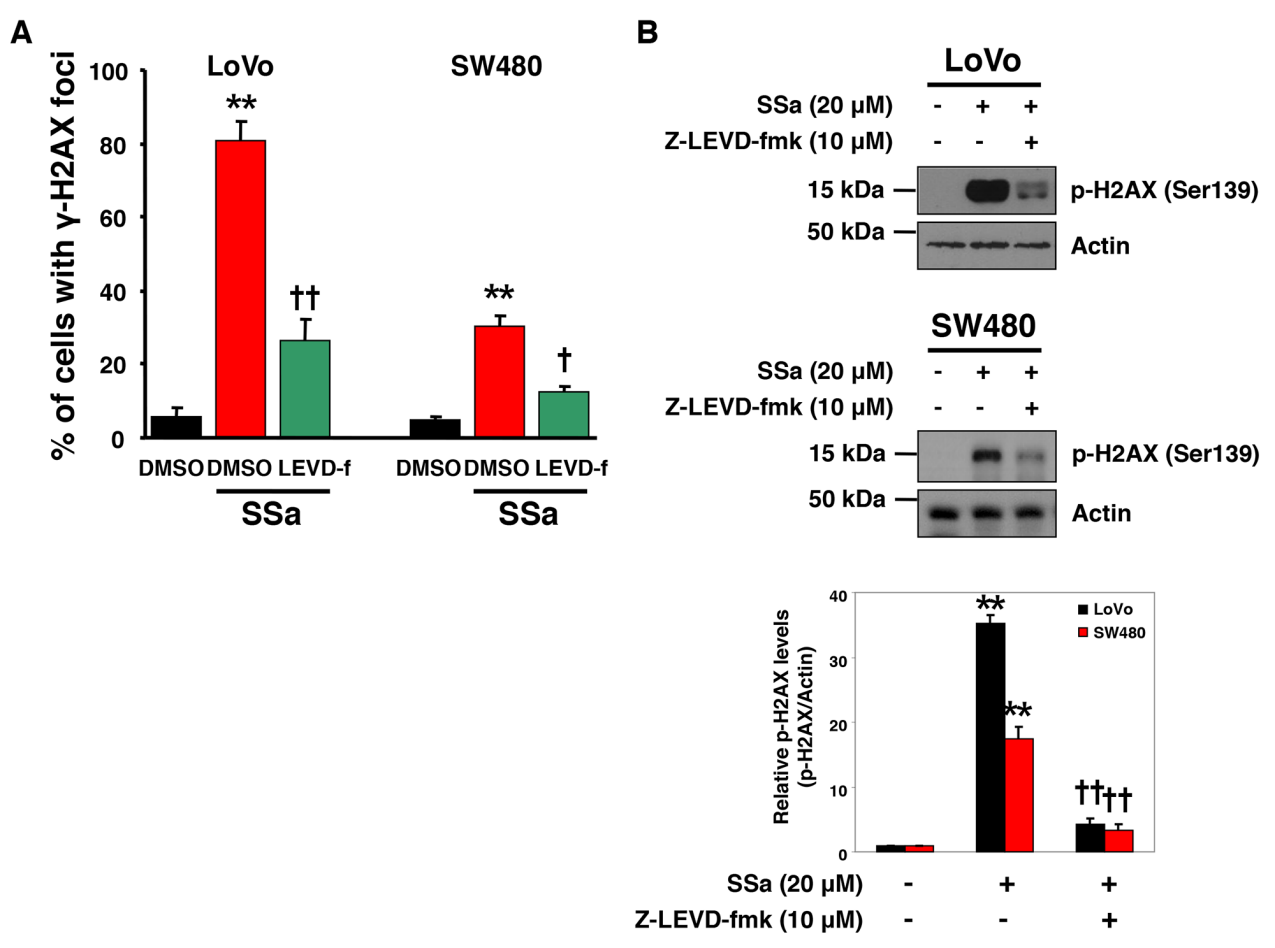
Caspase-4 plays a role in SSa-induced DNA damage response LoVo and SW480 cells were exposed to 20 μM SSa for 15 h in the presence or absence of z-LEVD-fmk (10 μM). **(A)** The percentage of cells positive for γ-H2AX foci was calculated as in Figure 5D. Results are mean ± standard error for three experiments. ^**^P < 0.01 compared with untreated controls. ^†^*P* < 0.05 and ^††^*P* < 0.01 compared with SSa alone. ANOVA and Tukey’s test were used for statistical analysis. **(B)** Western blotting was used to detect H2AX phosphorylation at Ser139. Blots are representative of three independent experiments, and data shown represent the mean ± standard error of three independent experiments. ^**^P < 0.01 compared with untreated controls. ^††^*P* < 0.01 compared with SSa alone. ANOVA and Tukey’s test were used for statistical analysis.

Considering the role of caspase-4 in SSa-induced caspase-2 activation and the role of caspase-2 in the DNA damage response, we investigated the role of caspase-4 in SSa-induced DNA damage in LoVo and SW480 cells. As shown in Figure 6A, γ-H2AX foci were increased markedly by SSa treatment, and these increases were suppressed significantly by the caspase-4 inhibitor z-LEVD-fmk. Western blot analysis using anti-phospho-H2AX antibody also showed that z-LEVD-fmk inhibited SSa-induced H2AX phosphorylation at Ser139 (Figure 6B). However, neither z-VDVAD-fmk, z-IETD-fmk nor z-DEVD-fmk inhibited SSa-induced H2AX phosphorylation at Ser139 (data not shown). These results indicate that caspase-4 may be a critical regulator of the DNA damage response in SSa-treated cells.

## DISCUSSION

Previously, we reported that SSa triggered apoptotic cell death in HCC cells through the sequential activation of caspase-2 and caspase-8 [14]. In this study, we expanded our findings to other cell types and demonstrated a critical role for caspase-4 in SSa-induced apoptosis and the DNA damage response. Because our previous results and other research have shown that SSa induced cancer cell death [14, 15, 17, 18], we first evaluated whether SSa cytotoxicity was universal. Cell viability assays showed that SSa cytotoxicity was cell type-dependent. Although colon cancer cells were very sensitive to SSa-triggered cytotoxicity, the majority of lung cancer, breast cancer, and leukemia cells were resistant to SSa. Moreover, at a clinically achievable concentration (2 mg/kg, once weekly), SSa significantly delayed colon cancer growth in a nude mouse xenograft model, supporting the possibility of a clinical application for SSa in colon cancer therapy.

ER stress is known to be a critical initiator and activator of cell death in pathological conditions [32-35]. ER stress, which results from the accumulation of unfolded or misfolded proteins in the ER lumen, has a profound effect on cancer cells. Therefore, pharmacological aggravation of ER stress has become an attractive strategy for cancer therapy. Potential anticancer agents, such as bortezomib, falcarindiol, curcumin analog, oplopantriol A, and WZ35, exert their activities by triggering ER stress [28, 36-39]. To investigate the possible effects of SSa on ER stress, the levels of various ER stress-associated proteins were assessed. PERK and PERK-induced phosphorylation of eIF2α initiates the early ER stress response and represses protein biosynthesis, preventing the influx of ER client proteins [40-42]. The transcription factor CHOP (C/EBP homologous protein) is activated by ER stress and is a component of the ER-mediated apoptosis pathway [43, 44]. Two ER-resident cysteine proteases (caspase-4 and caspase-12) are also activated by ER stress [1, 2]. There are conflicting reports on the role of these two caspases in ER stress-induced apoptosis. Some studies have shown that they are implicated in ER stress-induced apoptosis [1-3, 5-9]; however, at least one study has reported that these less recognized caspases are not required for ER stress-induced apoptosis [10]. SSa treatment did not trigger the phosphorylation of PERK or eIF2α in any of the colon cancer cell lines tested and increased CHOP expression in a cell type-dependent manner. Interestingly, SSa induced cleavage of caspase-4 and -12 and enzymatic activation of caspase-4 in all cell lines tested.

Recently, Flood et al. reported robust expression of caspase-4 and -5 within neoplastic tissue of colorectal tumors [45]. We examined the expression of caspase-4 in various cancer cells and found that caspase-4 expression was significantly higher in colon cancer cells than in other cancer cells (Supplementary Figure 10). Among lung cancer cells, caspase-4 was highly expressed in the SSa-sensitive H358 cell line. Caspase-4 expression was lowest in breast cancer cell lines compared to in other cell types tested. As an exception, some leukemia cells that are resistant to SSa treatment had high caspase-4 expression. Given the high basal expression of caspase-4 in colon cancer cells and given that SSa induces colon cancer-specific apoptosis and triggers caspase-4 cleavage and activation, we hypothesized that altered expression of caspase-4 may be an underlying cause of the selective effects of SSa. However, due to discrepancies between caspase-4 expression and SSa sensitivity, other possibilities cannot be excluded.

Another hypothesis we tested was whether the constitutive activation of NF-κB in HCC cells is a cause of the specific sensitivity of colon cancer cells to SSa-induced cytotoxicity. It has been reported that NF-κB is constitutively activated in most colon cancer cells [46]. Moreover, Din et al. reported that the effect of aspirin on NF-κB signaling is implicated in the specific sensitivity of colon cancer cells to aspirin-induced apoptosis [47]. We examined the effect of SSa on the NF-κB signaling pathway and found that SSa treatment induced significant IκBα (the NF-κB inhibitory protein) phosphorylation at Ser32 in SSa-sensitive colon cancer cells (LoVo, SW480, HT29, SW620) but not in SSa-resistant cells of other cancer types (H1299, Hs 578T, U937) (data not shown). Given that SSa-induced IκBα phosphorylation is generally restricted to colon cancer cells, we think that the sensitive response of the NF-κB pathway to SSa in colon cancer cells might be another underlying cause of the selective effects of SSa. Furthermore, it has been reported that enhancement of NF-κB activity increases the expression of caspase-4, both in terms of mRNA and protein [48]. Further studies into possible interactions between the effects of SSa on the NF-κB pathway and the effects of SSa on caspase-4 activation and apoptosis in cancer cells are in progress.

We assessed the role of caspase-4 in SSa-induced caspase activation and found inhibition of caspase-4 activation using z-LEVD-fmk (irreversible), Ac-LEVD-CHO (reversible), or RNA interference (siRNA), was associated with reduced activation of caspase-3, -8, and -2. However, inhibition of caspase-3, -8, and -2 using pharmacological inhibitors (z-DEVD-fmk, z-IETD-fmk, or z-VDVAD-fmk) or specific siRNAs did not lead to changes in caspase-4 activity. These findings suggest that caspase-4 activation is an important step in SSa-induced activation of caspase-2, -8 and -3. This is the first time caspase-2 has been identified as a novel substrate of caspase-4.

Even though caspase-4 and caspase-2 are typically classified as ‘inflammatory’ and ‘initiator’ caspases, respectively, these two proteins have similar domain structures. Both have caspase activation and recruitment domain (CARD domain), an interaction motif involved in processes related to inflammation and apoptosis [49, 50], in the N-terminal region. Since the CARD domain typically associates with CARD-containing proteins, this suggests that caspase-4 and caspase-2 have the potential to interact with each other. In addition, given that CARD-containing proteins are associated with immune responses, this suggests that SSa may also cause an immune response. Further studies are needed to investigate these issues.

We also assessed the role of caspase-4 in SSa-induced apoptosis using siRNA and the selective synthetic peptide caspase-4 inhibitors, z-LEVD-fmk and Ac-LEVD-CHO. Sub-G1 fraction analysis and Hoechst 33342 staining revealed that both caspase-4 inhibitors blocked the formation of sub-diploid DNA and apoptotic nuclei to a significant extent, indicating that caspase-4 is required for SSa-induced apoptosis. Moreover, siRNA knockdown of caspase-4 significantly decreased the numbers of hypodiploid cells and cells with condensed/fragmented nuclei, consistent with previous results. Clonogenicity is the degree to which a cell can form colonies in culture and is an indicator of the long-term survival of a cell. Interestingly, clonogenic assays showed that colony formation decreased for approximately 2 weeks following SSa treatment and was suppressed significantly by caspase-4 inhibition. Therefore, caspase-4 inhibition was correlated with enhanced clonogenic survival in colon cancer cells. However, caspase-12, another ER-resident caspase, did not seem to be involved in SSa-triggered apoptosis or clonogenic cell death, as evidenced by z-ATAD-fmk (caspase-12 inhibitor) not attenuating SSa-induced apoptosis or clonogenic cell death.

Caspase-12 also has a CARD domain. However, caspase-12 was not required for SSa-induced apoptosis; therefore, the CARD domain by itself does not explain the critical role of caspase-4 and/or the dispensable role of caspase-12 in SSa-induced apoptosis. Since initiator caspases activate other downstream caspases through their proteolytic activity, it is possible that the protease activity of caspase-4 is implicated in SSa-induced apoptosis and caspase activation. Similarly, human caspase-12 has no protease activity, even though it is highly related to members of the ICE subfamily.

We showed previously that SSa could activate caspase-2 activity [14]; as caspase-2 functions in response to DNA damage [19, 20], we also evaluated whether SSa induces DNA damage. CBMN, comet, and H2AX foci assays showed that SSa caused significant DNA damage in cancer cells. The tumor suppressor p53 protein primarily induces apoptosis in the DNA damage response. We assessed the expression of p53 and its apoptotic transcriptional target, bax after SSa treatment and found that SSa did not increase the expression of these proteins (data not shown). Furthermore, in our previous experiments using p53 wild-type HCT116 and p53^-/-^ HCT116 cells, we reported that the cytotoxic effect of SSa does not differ between these two cell types [14]. Collectively, these results indicate that SSa-induced cytotoxicity does not require p53 induction following DNA damage.

Given the role of caspase-4 in SSa-mediated caspase-2 activation and the role of caspase-2 in the DNA damage response, we investigated whether caspase-4 is involved in SSa-induced DNA damage. The formation of SSa-induced γ-H2AX foci, a marker of DNA damage, was reduced significantly after pretreatment with caspase-4 inhibitor. Because activated caspase-3 cleaves ICAD (inhibitor of caspase-activated DNase) and releases active CAD (caspase-activated DNase) from ICAD, which then cleaves DNA and provokes H2AX phosphorylation, the DNA damage we observed may be an effect rather than a cause of SSa-mediated apoptosis. To assess this possibility, we investigated whether pretreatment with inhibitors of caspase-3, as well as caspase-2 and -8, rescues SSa-induced DNA damage; none of these inhibitors were able to reduce SSa-induced H2AX phosphorylation (data not shown). Therefore, we hypothesize that SSa-induced DNA damage is a cause of SSa-induced apoptosis and that caspase-4 is an important regulator of this process. Further studies investigating linkage between the DNA damage response pathway and caspase-4 activation are warranted.

Taken together, these results suggest that sequential activation of caspase-4, -2, -8, and -3 is important for SSa-induced cancer cell apoptosis. Furthermore, caspase-4 plays an important role in the SSa-induced DNA damage response pathway and caspase-2 activation. Overall, our data suggest a model in which SSa induces colon cancer cell apoptosis via caspase-4 activation, followed by DNA damage and/or sequential activation of caspase-2, -8, and -3 (Supplementary Figure 11). Considering that SSa cytotoxicity is cell type-dependent, a tailor-made approach using SSa should be pursued in the development of cancer treatments.

## MATERIALS AND METHODS

### Reagents

Saikosaponin (SSa; Nacalai Tesque Inc., Kyoto, Japan) was dissolved in DMSO (Sigma Chemical Co., St. Louis, MO) to form a 10-mM stock solution and stored at –20°C until use. Propidium iodide (PI), RNase A, Hoechst 33342, cytochalasin-B, Giemsa stain, and 4’,6-diamidino-2-phenylindole (DAPI) were purchased from Sigma. Caspase colorimetric assay kits were purchased from BioVision (Palo Alto, CA). Caspase-4 inhibitors, z-LEVD-fmk and Ac-LEVD-CHO, were obtained from Enzo Life Sciences (Plymouth Meeting, PA). The caspase-12 inhibitor, z-ATAD-fmk, was obtained from BioVision. z-DEVD-fmk (caspase-3 inhibitor), z-VDVAD-fmk (caspase-2 inhibitor), and z-IETD-fmk (caspase-8 inhibitor) were obtained from Calbiochem (La Jolla, CA). The annexin V-fluorescein isothiocyanate (FITC) apoptosis detection kit was purchased from BD Biosciences (Franklin Lakes, NJ, USA).

### Cell culture

Human colon carcinoma (HCC; HCT116, LoVo, HT-29, SW48, SW480, and SW620), human lung cancer (H460, A549, H520, H358, and H1299), human breast cancer (BT20, Hs 578T, MCF7, and MDAMB231), and human leukemia cell lines (HL-60, Jurkat, MOLT-4, U937, and K562) were obtained from the American Type Culture Collection (ATCC, Manassas, VA). Cells were grown in DMEM or RPMI 1640 medium supplemented with 10% fetal bovine serum (FBS), 100 U/mL penicillin, and 100 μg/mL streptomycin (all from GIBCO, Invitrogen, Carlsbad, CA). The cultures were maintained at 37°C under an atmosphere of 5% CO_2_.

### Treatment with SSa and caspase inhibitors

For caspase activity assays, SW480 cells were pretreated with caspase inhibitors (z-DEVD-fmk, z-VDVAD-fmk, z-IETD-fmk, or z-LEVD-fmk; 10 μM) or vehicle (DMSO) for 1 h, and then treated with 20 μM SSa. For apoptosis and colony formation assays, two HCC cell lines (LoVo and SW480) were pretreated with caspase-4 inhibitors (z-LEVD-fmk or Ac-LEVD-CHO; 10 μM), caspase-12 inhibitor (z-ATAD-fmk; 10 μM), or vehicle (DMSO for z-LEVD-fmk and z-ATAD-fmk or DDW for Ac-LEVD-CHO) for 1 h and then treated with 20 μM SSa. The time of SSa treatment is given in each figure legend.

### Cell viability assay

Following SSa treatment, the rate of cell survival was determined using a Trypan Blue exclusion assay. In this assay, viable cells exclude Trypan Blue dye. HCC (HCT116, LoVo, HT-29, SW48, SW480, and SW620), human lung cancer (H460, A549, H520, H358, and H1299), human breast cancer (BT20, Hs 578T, MCF7, and MDAMB231), and human leukemia (HL-60, Jurkat, MOLT-4, U937, and K562) cell lines were treated with 20 μM SSa for up to 40 h. Prior to SSa treatment, cell viability was verified by trypan blue exclusion and was routinely over 97%. The observed percentage of live cells is indicated in each Figure.

### Tumor growth-delay assay

Flank xenografts were established in 6-week-old female athymic BALB/c nude mice by subcutaneous injection of 2×10^6^ LoVo and SW480 cells resuspended in 0.1 ml of PBS. Tumors were allowed to grow for 7 days before treatment. The mice were randomized into two groups (DMSO vehicle treatment and SSa treatment) of five mice each. SSa was solubilized in 10% DMSO in PBS, pH 7.4, immediately before each treatment, and SSa or the vehicle was administered to mice by intraperitoneal injection at a dose of 2 mg/kg once per week. Tumors were measured every 3 days with calipers in a blind fashion, and tumor volume was calculated as the largest diameter (mm) × smallest diameter^2^ (mm^2^)/2. Mice were treated only after obtaining approval from the Yonsei University Animal Care and Use Committee, and experiments were carried out according to relevant guidelines.

### Quantitative RT-PCR assay for detection of the mRNA of ER stress-induced genes

Total RNA was isolated from HCC cells using an RNeasy Mini Kit (Qiagen) according to the manufacturer’s instructions. cDNAs were synthesized using the iScript^™^ cDNA synthesis kit (Bio-Rad). To determine mRNA levels of PERK, CHOP, ATF4, and XBP1, quantitative RT-PCR was performed on a Mastercycler ep realplex thermal cycler (Eppendorf), in a total volume of 20 μl containing 200 nM primers and 1× ABsolute Blue QPCR SYBR Green Mix (ABgene). For human PERK amplification, primers hPERK-F (5’-ATGAGACAGAGTTGCGACCG-3’) and hPERK-R (5’-TGCTAAGGCTGGATGACACC-3’) were used. For human CHOP amplification, primers hCHOP-F (5’-ACAGAGTGGTCATTCCCCAGCC-3’) and hCHOP-R (5’-TTCGGTCAATCAGAGCTCGGCG-3’) were used. For human ATF4 amplification, primers hATF4-F (5’-GCCAAGCACTTCAAACCTC-3’) and hATF4-R (5’-ATCTATACCCAACAGGGCATC-3’) were used. For human XBP1 amplification, primers hXBP1-F (5’-CAGAGATCGAAAGAAGGCTC-3’) and hXBP1-R (5’-CAAGCGCTGTCTTAACTCC-3’) were used. For human GAPDH amplification, primers hHPRT-F (5’-TGGCGTCGTGATTAGTGATG-3’) and hHPRT-R (5’-GCTACAATGTGATGGCCTCC-3’) were used.

### Assessment of caspase activity

Caspase colorimetric assay kits (BioVision) were used to determine the enzymatic activities of caspases (caspase-3, -4, -2, and -8). Cell lysates were prepared in the lysis buffer provided by the manufacturer. Lysates were normalized for protein content and then incubated with labeled substrates at 37°C for 2 h. Caspase activity was measured by spectrophotometric detection of the chromophore p-nitroanilide (pNA) at 405 nm after cleavage from the substrate.

### siRNA transfection targeting caspase-4

LoVo and SW480 cells, which were ∼30% confluent on the day of transfection, were transiently transfected with small interfering RNA (siRNA) against caspase-4 using Lipofectamine RNAiMAX Reagent (Invitrogen) according to the manufacturer’s protocol. Control treated cells were exposed only to the transfection reagent, and negative control cells were transfected with scrambled siRNA against caspase-4. Thirty hours after transfection, cells were challenged with 20 μM SSa. The caspase-4 siRNA sequence was ACAGCTGTTGTTGAGCGAA, and the scrambled siRNA sequence was GAUCAUACGUGCGAUCAGA. All siRNAs were designed using Rosetta with a proprietary algorithm.

### Flow cytometric detection of the sub-G1 fraction

LoVo and SW480 cells were treated with various concentrations of SSa for 30 h in the presence or absence of caspase-4 inhibitors (z-LEVD-fmk or Ac-LEVD-CHO), caspase-12 inhibitor (z-ATAD-fmk), or caspase-4 siRNA. Cellular DNA was stained with PI and quantified by sub-G1 fluorescence-activated cell sorting (FACS) analysis using a FACScan flow cytometer (Becton Dickinson, Franklin Lakes, NJ). Cells were fixed in ice-cold 70% ethanol, washed twice with PBS, and then stained with 50 ithmL PI and 100 μg/mL RNase A. The cell suspension was incubated in the dark at room temperature for 30 min, and 10,000 cells were measured per sample. Analyses were performed using Cell Quest Pro software (Becton Dickinson).

### Assessment of apoptotic nuclear morphology

LoVo and SW480 cells were treated with various concentrations of SSa for 24 h in the presence or absence of caspase-4 inhibitors (z-LEVD-fmk or Ac-LEVD-CHO), caspase-12 inhibitor (z-ATAD-fmk), or caspase-4 siRNA. Following fixation in 4% paraformaldehyde in PBS for 10 min at room temperature, cells were incubated in 2 μg/mL Hoechst 33342 for 30 min in the dark. More than 200 cells were observed under a fluorescence microscope and assessed for morphological features of apoptosis. Apoptotic nuclei were identified by the presence of condensed chromatin around the periphery of the nuclear membrane or totally fragmented nuclear bodies.

### Assessment of annexin V/PI double staining

LoVo and SW480 cells were treated with various concentrations of SSa for 24 h. Cells were stained with annexin V-FITC and PI (both from BD Biosciences) for 15 min at room temperature in annexin V binding buffer, then analyzed using flow cytometry (Becton Dickinson, Franklin Lakes, NJ). Emissions from annexin V and PI were detected in the FL-1 and FL-2 channels, respectively. For each sample, data from 10,000 cells were recorded in a list using logarithmic scales. Data analysis was conducted using Cell Quest Pro software (Becton Dickinson). Annexin V positive results indicate apoptotic cells.

### Colony formation assay

LoVo and SW480 cells were trypsinized, counted, and plated into 60-mm dishes at a density of 500 cells/dish. Cells were treated with 15 μM SSa for 12–14 d to allow for colony formation in the presence or absence of caspase-4 inhibitors (z-LEVD-fmk or Ac-LEVD-CHO). After incubation, colonies were fixed and stained with 1% methylene blue in 50% ethanol at room temperature, and colonies consisting of more than 100 cells per plate were counted.

### Cytokinesis-block micronucleus (CBMN) assay

LoVo and SW480 cells were treated with 20 μM SSa for up to 15 h and then with cytochalasin-B (4 μg/mL; Sigma) for 28 h to inhibit cell division. Cytokinesis-block micronucleus (CBMN) preparations were prepared according to a modified version of the method developed by Fenech and Morley [51]. Cells were harvested for slide preparation, fixed in methanol for 15 min, air-dried, and then stained in 5% Giemsa solution. After staining, the slides were rinsed in water, air-dried, and mounted with cover slips. Slides were examined with a Leica DMLB optical microscope (Leica Microsystems AG, Wetzlar, Germany) at 40× magnification. Scoring was conducted based on the criteria established by Fenech [52]. In total, 1000 binucleated cells were scored. All slides were coded and scored blindly.

### Single-cell gel electrophoresis (SCGE, comet) assay

LoVo and SW480 cells were treated with 20 μM SSa for up to 15 h. After treatment, neutral [for double-strand breaks (DSBs)] and alkaline [for single-strand breaks (SSBs) and DSBs] single-cell gel electrophoresis (SCGE, comet) assays were performed using a Trevigen comet assay kit (Trevigen, Inc., Gaithersburg, MD). Cells were mixed with molten low-melting point agarose at 37°C at a 1:10 (v/v) ratio and spread immediately onto frosted microscope slides. The slides were incubated at 4°C for 20 min and then immersed in lysis solution at 4°C for 1 h. For the alkaline assay, slides were immersed in freshly prepared alkaline unwinding solution (pH>13) at 4°C for 1 h, followed by alkaline electrophoresis for 30 min at 21 V (1 V/cm). After electrophoresis, slides were washed twice by immersion in distilled water for 10 min, followed by immersion in 70% ethanol for 5 min. For the neutral assay, slides were immersed in neutral electrophoresis buffer at 4°C for 30 min, followed by neutral electrophoresis for 45 min at 21 V (1 V/cm). After electrophoresis, slides were immersed in DNA precipitation solution for 30 min at room temperature, then in 70% ethanol for 30 min. DNA was stained with SYBR Green and examined using Komet 5.5 imaging software. The Olive tail moment (OTM), defined as the product of the percentage of DNA in the tail and the displacement between the position of the mean center of mass in the heads and tails, was used as a measure of DNA damage. Two hundred comets were evaluated per sample.

### Immunostaining for γ-H2AX foci

LoVo and SW480 cells were treated with 20 μM SSa for 15 h in the presence or absence of z-LEVD-fmk (caspase-4 inhibitor), then fixed in 4% paraformaldehyde at 4°C for 15 min. The fixed cells were permeabilized in PBS containing 100 mM Tris-HCl, 50 mM ethylenediamine tetra-acetic acid (EDTA), and 0.5% Triton X-100 for 15 min at room temperature. Cells were incubated with phospho-H2AX (ab2893; Abcam, Cambridge, UK) primary antibody diluted to a 1:100 ratio at 4°C overnight and then detected with AlexaFluor 594-conjugated anti-rabbit secondary antibody (A-11037; Molecular Probes, Eugene, OR, USA). Slides were counterstained with DAPI, and images were acquired on an ECLIPSE E600 fluorescence microscope (Nikon, Tokyo, Japan). Cells were classified as ‘positive’ when more than three H2AX foci were observed per cell.

### Immunoblot analysis

Cells were solubilized in lysis buffer [50 mM Tris-HCl (pH 7.4), 150 mM NaCl, 1 mM EDTA, 1% Triton X-100, 1% sodium deoxycholate, 0.1% SDS, and 1 mM DTT] containing protease and phosphatase inhibitor cocktails (both from Sigma). Following centrifugation, the concentration of protein in the soluble faction was determined using the Bradford assay (Bio-Rad Laboratories, Hercules, CA). Protein samples were loaded into a SDS-polyacrylamide gel (10 μg/lane), separated by SDS-polyacrylamide gel electrophoresis (SDS-PAGE), transferred onto polyvinylidene fluoride (PVDF) membrane (NEN, PerkinElmer, Wellesley, MA), and probed with specific antibodies against the following proteins (the epitope and catalog numbers are listed in parentheses): p-PERK (Thr981) (sc-32577), cleaved-caspase-4 (sc-22174; Santa Cruz Biotechnology, Santa Cruz, CA); p-eIF2α (Ser51) (9721), CHOP (2895), pro-caspase-4 (4450), caspase-2 (2224), caspase-8 (9746), p-H2AX (Ser139) (2577), actin (3700; Cell Signaling Technology, Danvers, MA, USA); t-Bid (44-433G; Biosource, Bethesda, MD), and pro-caspase-12 (ab62484; Abcam, Cambridge, UK). Primary antibodies were detected using horseradish peroxidase-conjugated secondary antibodies and visualized using a Western Lightning Plus-ECL (Perkin Elmer, Waltham, MA) chemiluminescence system. The results of immunoblotting were quantified by densitometry and analyzed using ImageQuant software. Data were normalized for actin levels and expressed using arbitrary units.

### Statistical analysis

Statistical analyses were performed using SAS 8.1 software for Windows (SAS Institute, Inc., Cary, NC). All experiments were conducted in triplicate and results are presented as the mean ± standard error. Data were evaluated using the Mann-Whitney *U* test for comparison between two independent groups or one-way analysis of variance (ANOVA) with Tukey’s *post hoc* test for multiple comparisons. *P* < 0.05 was considered statistically significant, and *P* < 0.01 was considered highly statistically significant.

## SUPPLEMENTARY MATERIALS FIGURES



## References

[1] Nakagawa T, Zhu H, Morishima N, Li E, Xu J, Yankner BA, Yuan JY (2000). Caspase-12 mediates endoplasmic-reticulum-specific apoptosis and cytotoxicity by amyloid-beta. Nature.

[2] Hitomi J, Katayama T, Eguchi Y, Kudo T, Taniguchi M, Koyama Y, Manabe T, Yamagishi S, Bando Y, Imaizumi K, Tsujimoto Y, Tohyama M (2004). Involvement of caspase-4 in endoplasmic reticulum stress-induced apoptosis and A beta-induced cell death. J Cell Biol.

[3] Hoppe V, Hoppe J (2004). Mutations dislocate caspase-12 from the endoplasmatic reticulum to the cytosol. FEBS Lett.

[4] Sollberger G, Strittmatter GE, Kistowska M, French LE, Beer HD (2012). Caspase-4 is required for activation of inflammasomes. J Immunol.

[5] Kamada S, Washida M, Hasegawa J, Kusano H, Funahashi Y, Tsujimoto Y (1997). Involvement of caspase-4(-like) protease in Fas-mediated apoptotic pathway. Oncogene.

[6] Mao ZG, Jiang CC, Yang F, Thorne RF, Hersey P, Zhang XD (2010). TRAIL-induced apoptosis of human melanoma cells involves activation of caspase-4. Apoptosis.

[7] Kim SJ, Zhang ZJ, Hitomi E, Lee YC, Mukherjee AB (2006). Endoplasmic reticulum stress-induced caspase-4 activation mediates apoptosis and neurodegeneration in INCL. Hum Mol Genet.

[8] Bian ZM, Elner SG, Elner VM (2009). Dual involvement of caspase-4 in inflammatory and er stress-induced apoptotic responses in human retinal pigment epithelial cells. Invest Ophthalmol Vis Sci.

[9] Rosati E, Sabatini R, Rampino G, De Falco F, Di Ianni M, Falzetti F, Fettucciari K, Bartoli A, Screpanti I, Marconi P (2010). Novel targets for endoplasmic reticulum stress-induced apoptosis in B-CLL. Blood.

[10] Obeng EA, Boise LH (2005). Caspase-12 and caspase-4 are not required for caspase-dependent endoplasmic reticulum stress-induced apoptosis. J Biol Chem.

[11] Wu WS, Hsu HY (2001). Involvement of p-15(INK4b) and p-16(INK4a) gene expression in saikosaponin a and TPA-induced growth inhibition of HepG2 cells. Biochem Biophys Res Commun.

[12] Sun Y, Cai TT, Zhou XB, Xu Q (2009). Saikosaponin a inhibits the proliferation and activation of T cells through cell cycle arrest and induction of apoptosis. Int Immunopharmacol.

[13] Wang QO, Zheng XL, Yang L, Shi F, Gao LB, Zhong YJ, Sun H, He F, Lin Y, Wang X (2010). Reactive oxygen species-mediated apoptosis contributes to chemosensitization effect of saikosaponins on cisplatin-induced cytotoxicity in cancer cells. J Exp Clin Cancer Res.

[14] Kim BM, Hong SH (2011). Sequential caspase-2 and caspase-8 activation is essential for saikosaponin a-induced apoptosis of human colon carcinoma cell lines. Apoptosis.

[15] Qian L, Murakami T, Kimura Y, Takahashi M, Okita K (1995). Saikosaponin a-induced cell-death of a human hepatoma-cell line (Huh-7) - the significance of the sub-G(1) peak in a DNA histogram. Pathol Int.

[16] Wu WS (2003). ERK signaling pathway is involved in p15(INK4b)/p16(INK4a) expression and HepG2 growth inhibition triggered by TPA and saikosaponin a. Oncogene.

[17] Chen JC, Chang NW, Chung JG, Chen KC (2003). Saikosaponin-A induces apoptotic mechanism in human breast MDA-MB-231 and MCF-7 cancer cells. Am J Chin Med.

[18] Lee KJ, Xu MY, Shehzad O, Seo EK, Kim YS (2014). Separation of triterpenoid saponins from the root of Bupleurum falcatum by counter current chromatography: the relationship between the partition coefficients and solvent system composition. J Sep Sci.

[19] Zhivotovsky B, Orrenius S (2005). Caspase-2 function in response to DNA damage. Biochem Biophys Res Commun.

[20] Kumar S (2009). Caspase 2 in apoptosis, the DNA damage response and tumour suppression: enigma no more?. Nat Rev Cancer.

[21] Dorstyn L, Puccini J, Wilson CH, Shalini S, Nicola M, Moore S, Kumar S (2012). Caspase-2 deficiency promotes aberrant DNA-damage response and genetic instability. Cell Death Diff.

[22] Robertson JD, Enoksson M, Suomela M, Zhivotovsky B, Orrenius S (2002). Caspase-2 acts upstream of mitochondria to promote cytochrome c release during etoposide-induced apoptosis. J Biol Chem.

[23] Panaretakis T, Laane E, Pokrovskaja K, Bjorklund AC, Moustakas A, Zhivotovsky B, Heyman M, Shoshan MC, Grander D (2005). Doxorubicin requires the sequential activation of caspase-2, protein kinase C delta, and c-Jun NH2-terminal kinase to induce apoptosis. Mol Biol Cell.

[24] Wejda M, Impens F, Takahashi N, Van Damme P, Gevaert K, Vandenabeele P (2012). Degradomics reveals that cleavage specificity profiles of caspase-2 and effector caspases are alike. J Biol Chem.

[25] Wong VK, Li T, Law BY, Ma ED, Yip NC, Michelangeli F, Law CK, Zhang MM, Lam KY, Chan PL, Liu L (2013). Saikosaponin-d, a novel SERCA inhibitor, induces autophagic cell death in apoptosis-defective cells. Cell Death Dis.

[26] Kim BM, Maeng K, Lee KH, Hong SH (2011). Combined treatment with the Cox-2 inhibitor niflumic acid and PPARgamma ligand ciglitazone induces ER stress/caspase-8-mediated apoptosis in human lung cancer cells. Cancer Lett.

[27] Upton JP, Austgen K, Nishino M, Coakley KM, Hagen A, Han D, Papa FR, Oakes SA (2008). Caspase-2 cleavage of BID is a critical apoptotic signal downstream of endoplasmic reticulum stress. Mol Cell Biol.

[28] Gu HT, Chen XQ, Gao GX, Dong HJ (2008). Caspase-2 functions upstream of mitochondria in endoplasmic reticulum stress-induced apoptosis by bortezomib in human myeloma cells. Mol Cancer Ther.

[29] Rogakou EP, Pilch DR, Orr AH, Ivanova VS, Bonner WM (1998). DNA double-stranded breaks induce histone H2AX phosphorylation on serine 139. J Biol Chem.

[30] Burma S, Chen BP, Murphy M, Kurimasa A, Chen DJ (2001). ATM phosphorylates histone H2AX in response to DNA double-strand breaks. J Biol Chem.

[31] Sedelnikova OA, Pilch DR, Redon C, Bonner WM (2003). Histone H2AX in DNA damage and repair. Cancer Biol Ther.

[32] Kim I, Xu WJ, Reed JC (2008). Cell death and endoplasmic reticulum stress: disease relevance and therapeutic opportunities. Nat Rev Drug Discov.

[33] Yang CX, DiIorio P, Jurczyk A, O’Sullivan-Murphy B, Urano F, Bortell R (2013). Pathological endoplasmic reticulum stress mediated by the IRE1 pathway contributes to pre-insulitic beta cell apoptosis in a virus-induced rat model of type 1 diabetes. Diabetologia.

[34] Sano R, Reed JC (2013). ER stress-induced cell death mechanisms. Biochim Biophys Acta.

[35] Oakes SA, Papa FR (2015). The role of endoplasmic reticulum stress in human pathology. Ann Rev Pathol.

[36] Jin HR, Zhao J, Zhang Z, Liao Y, Wang CZ, Huang WH, Li SP, He TC, Yuan CS, Du W (2012). The antitumor natural compound falcarindiol promotes cancer cell death by inducing endoplasmic reticulum stress. Cell Death Dis.

[37] Liu ZG, Sun YS, Ren LQ, Huang Y, Cai YP, Weng QY, Shen XQ, Li XK, Liang G, Wang Y (2013). Evaluation of a curcumin analog as an anti-cancer agent inducing ER stress-mediated apoptosis in non-small cell lung cancer cells. BMC Cancer.

[38] Jin HR, Liao Y, Li X, Zhang Z, Zhao J, Wang CZ, Huang WH, Li SP, Yuan CS, Du W (2014). Anticancer compound Oplopantriol A kills cancer cells through inducing ER stress and BH3 proteins Bim and Noxa. Cell Death Dis.

[39] Zou P, Zhang JR, Xia YQ, Kanchana K, Guo GL, Chen WB, Huang Y, Wang Z, Yang SL, Liang G (2015). ROS generation mediates the anti-cancer effects of WZ35 via activating JNK and ER stress apoptotic pathways in gastric cancer. Oncotarget.

[40] Koumenis C, Naczki C, Koritzinsky M, Rastani S, Diehl A, Sonenberg N, Koromilas A, Wouters BG (2002). Regulation of protein synthesis by hypoxia via activation of the endoplasmic reticulum kinase PERK and phosphorylation of the translation initiation factor eIF2 alpha. Mol Cell Biol.

[41] Owen CR, Kumar R, Zhang P, McGrath BC, Cavener DR, Krause GS (2005). PERK is responsible for the increased phosphorylation of eIF2 alpha and the severe inhibition of protein synthesis after transient global brain ischemia. J Neurochem.

[42] Teske BF, Wek SA, Bunpo P, Cundiff JK, McClintick JN, Anthony TG, Wek RC (2011). The eIF2 kinase PERK and the integrated stress response facilitate activation of ATF6 during endoplasmic reticulum stress. Mol Biol Cell.

[43] Oyadomari S, Mori M (2004). Roles of CHOP/GADD153 in endoplasmic reticulum stress. Cell Death Diff.

[44] Szegezdi E, Logue SE, Gorman AM, Samali A (2006). Mediators of endoplasmic reticulum stress-induced apoptosis. EMBO Rep.

[45] Flood B, Oficjalska K, Laukens D, Fay J, O’Grady A, Caiazza F, Heetun Z, Mills KH, Sheahan K, Ryan EJ, Doherty GA, Kay E, Creagh EM (2015). Altered expression of caspases-4 and -5 during inflammatory bowel disease and colorectal cancer: Diagnostic and therapeutic potential. Clin Exp Immunol.

[46] Din FV, Dunlop MG, Stark LA (2004). Evidence for colorectal cancer cell specificity of aspirin effects on NF kappa B signalling and apoptosis. Br J Cancer.

[47] Hassanzadeh P (2011). Colorectal cancer and NF-kappaB signaling pathway. Gastroenterol Hepatol Bed Bench.

[48] Yang HJ, Wang M, Wang L, Cheng BF, Lin XY, Feng ZW (2015). NF-kappa B regulates caspase-4 expression and sensitizes neuroblastoma cells to fas-induced apoptosis. PLoS One.

[49] Park HH, Lo YC, Lin SC, Wang L, Yang JK, Wu H (2007). The death domain superfamily in intracellular signaling of apoptosis and inflammation. Ann Rev Immunol.

[50] Inohara N, Nunez G (2003). NODs: intracellular proteins involved in inflammation and apoptosis. Nat Rev Immunol.

[51] Fenech M, Morley AA (1985). Measurement of micronuclei in lymphocytes. Mutat Res.

[52] Fenech M (2000). The *in vitro* micronucleus technique. Mutat Res.

