# Supplementary material for: Caspase-4 is essential for saikosaponin a-induced apoptosis acting upstream of caspase-2 and γ-H2AX in colon cancer cells
Source: Oncotarget. 2017 Nov 1;8(59):100433–48. doi: 10.18632/oncotarget.22247 (PMC5725032; doi:10.18632/oncotarget.22247)
Supplement: Supplementary file 1 [file oncotarget-08-100433-s001.pdf]

Caspase-4 is essential for saikosaponin a-induced apoptosis acting upstream of caspase-2 and γ-H2AX in colon cancer cells

SUPPLEMENTARY MATERIALS

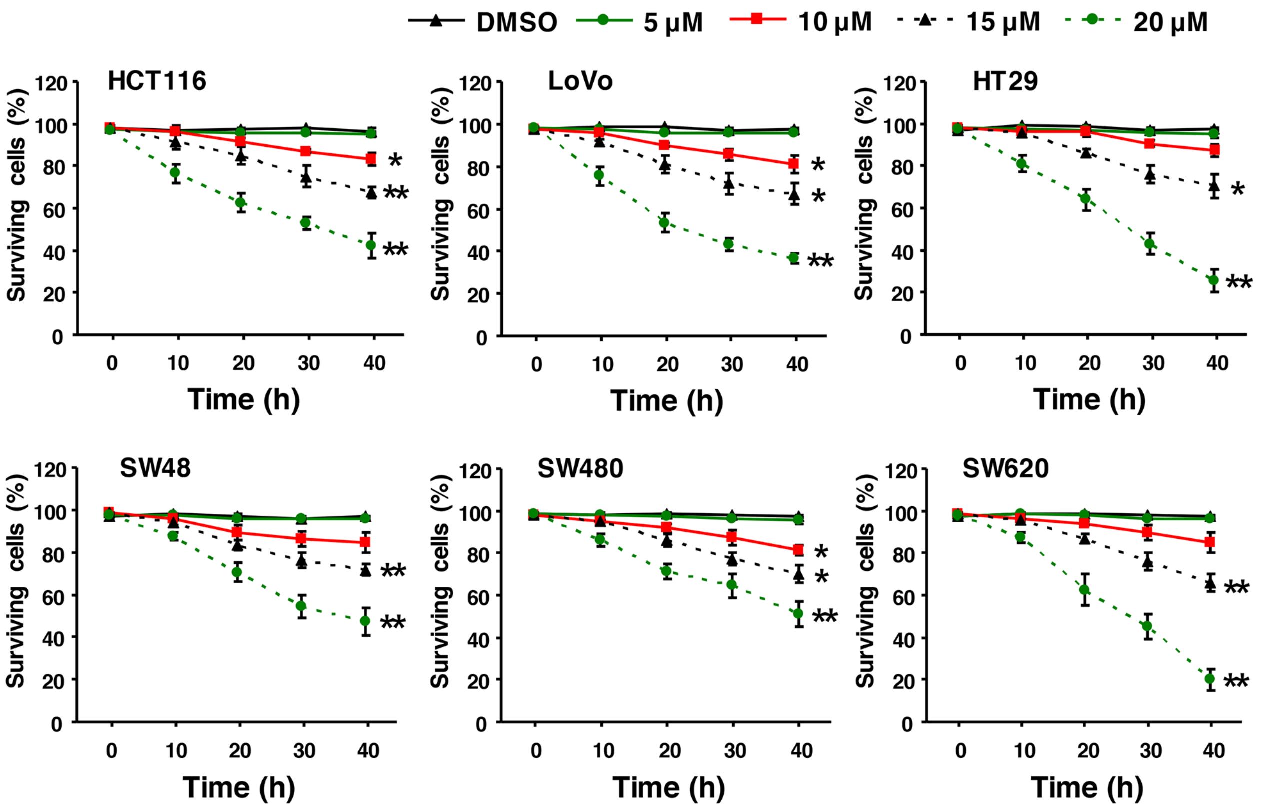

Supplementary Figure 1: Dose- and time-dependent effects of SSa on the viability of human HCC cells. HCT116, LoVo, HT29, SW48, SW480, and SW620 cells were challenged with the indicated concentrations of SSa for up to 40 h. Results are the mean ± standard error from three experiments. $^{*}P < 0.05$ and $^{**}P < 0.01$ compared with DMSO control. The Mann-Whitney U test was used for statistical analysis.

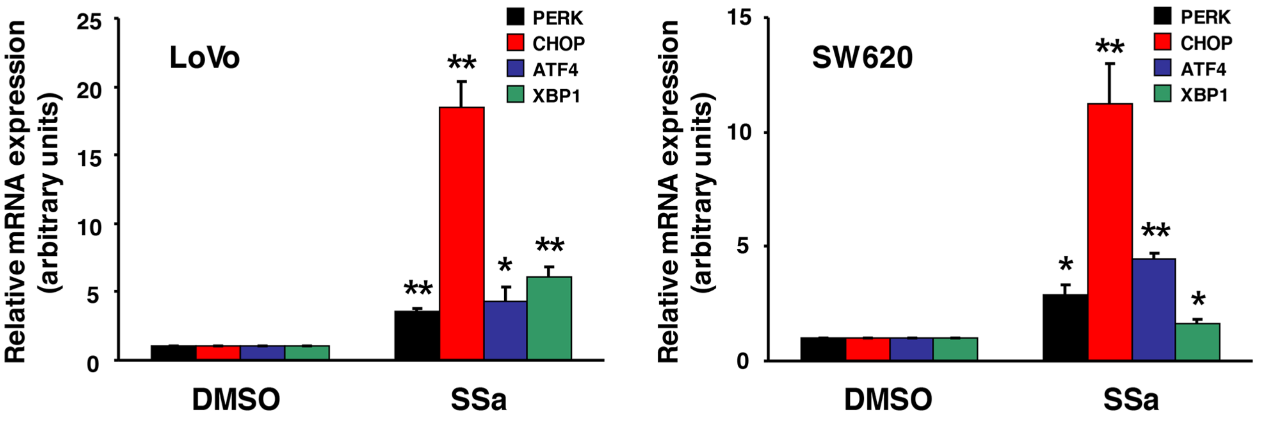

Supplementary Figure 2: Effect of SSa on *PERK*, *CHOP*, *ATF4*, and *XBP1* mRNA expression in LoVo and SW620 cells. Equal amounts of cell lysates were subjected to quantitative RT-PCR. *GAPDH* was used as an invariant endogenous control. Fold changes in mRNA levels of target genes relative to *GAPDH* were calculated. All samples were measured in triplicate. Each column represents the mean ± standard error of three independent experiments. $^{*}P < 0.05$ and $^{**}P < 0.01$ compared with DMSO control. The Mann-Whitney *U* test was used for statistical analysis.

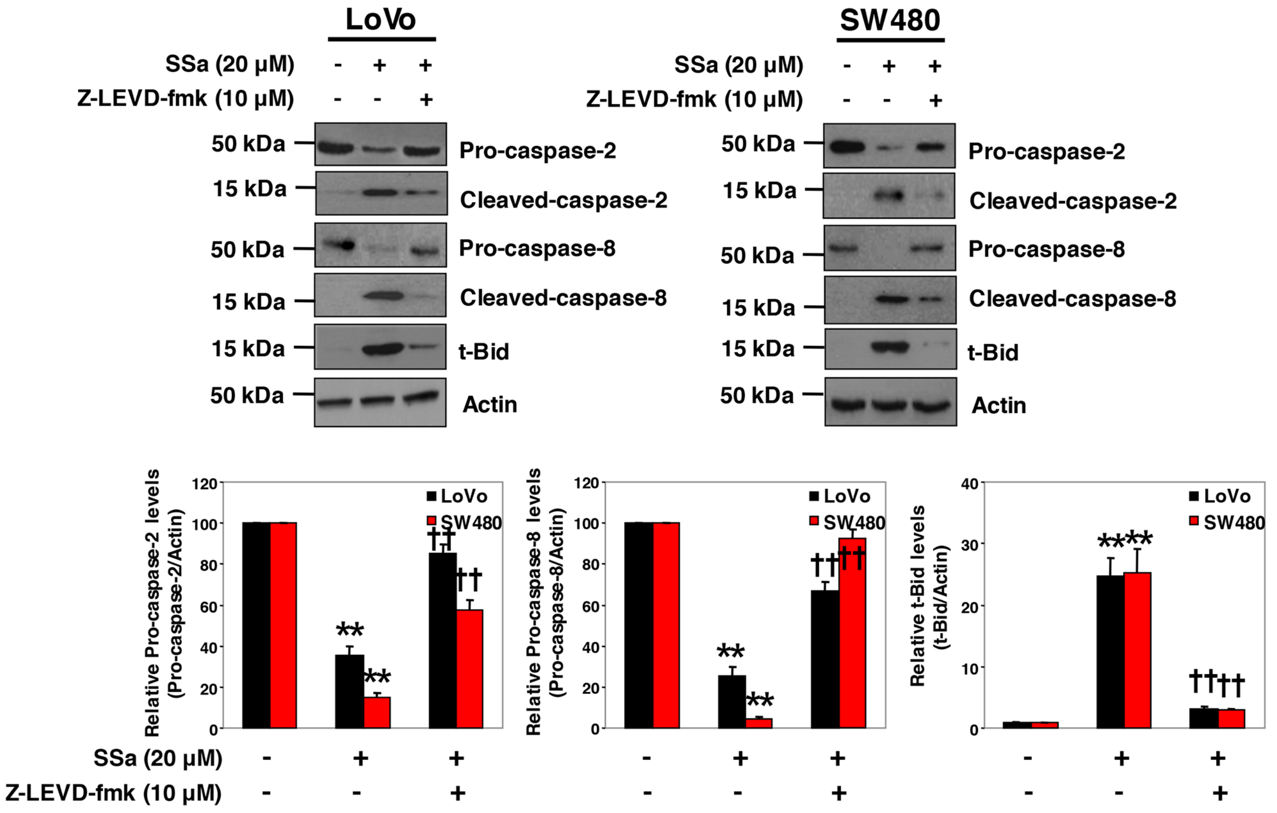

Supplementary Figure 3: Involvement of caspase-4 in SSa-induced cleavage of caspase-2, -8, and Bid. LoVo and SW480 HCC cells were exposed to 20 μM SSa for 15 h in the presence or absence of the caspase-4 inhibitor, z-LEVD-fmk (10 μM). Caspase-2, -8, and truncated Bid (t-Bid) levels were detected by western blot. Blots are representative of three independent experiments, and data shown represent mean ± standard error of three independent experiments. $^{**}P < 0.01$ compared with untreated controls. $^{††}P < 0.01$ compared with SSa alone. ANOVA and Tukey's test were used for statistical analysis.

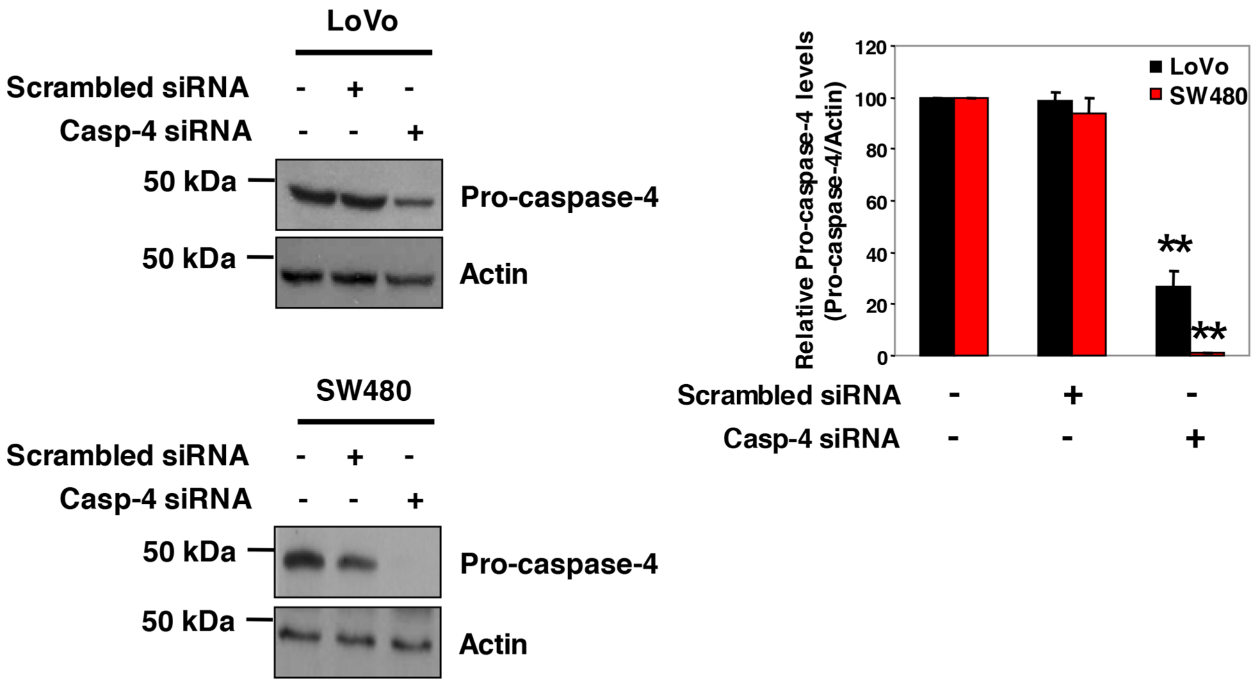

Supplementary Figure 4: Knockdown efficiencies of caspase-4 protein levels after transfection with siRNA specific for caspase-4 for 30 h. Protein levels were assessed by western blot. Blots are representative of three independent experiments, and data shown represent mean ± standard error of three independent experiments. $^{**}P < 0.01$ compared scrambled siRNA-treated cells. The Mann-Whitney *U* test was used for statistical analysis.

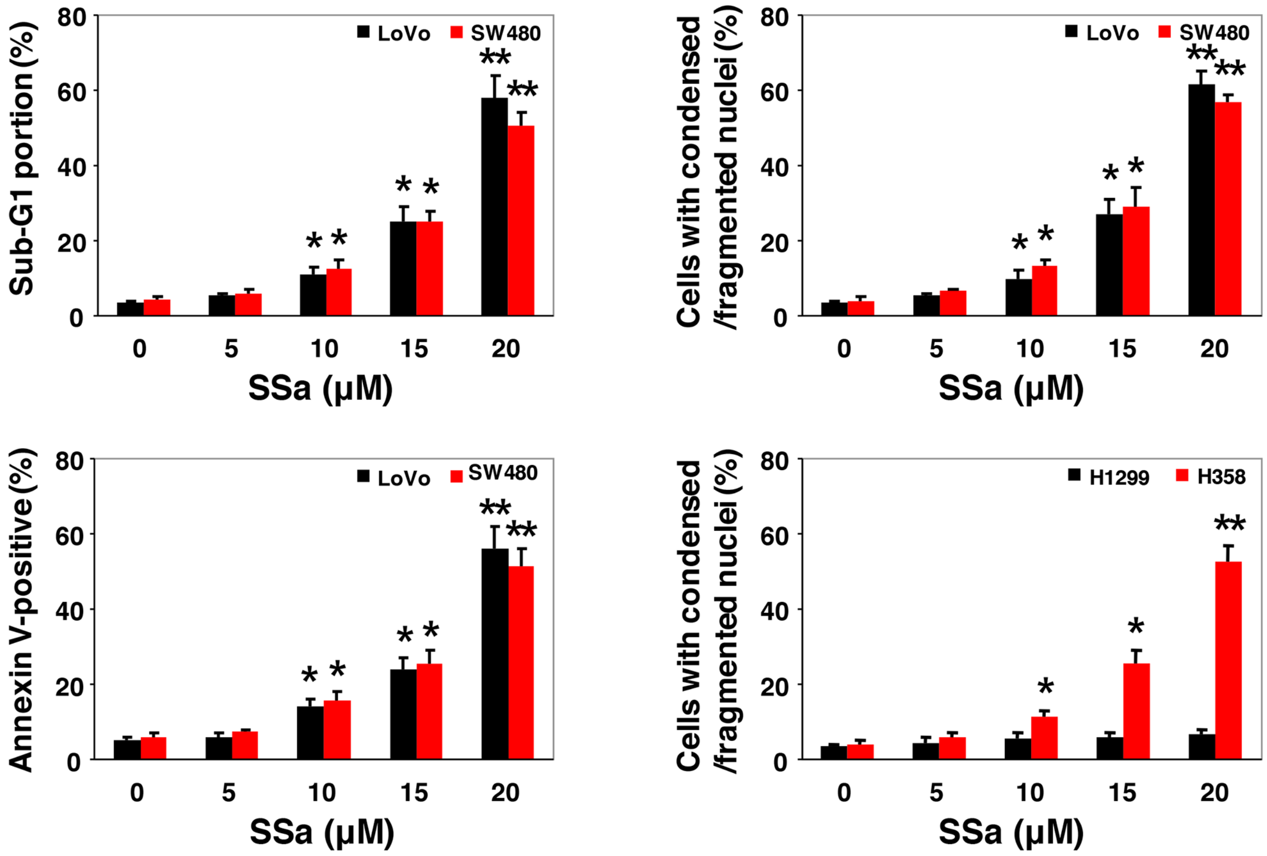

Supplementary Figure 5: Induction of apoptosis by SSa in HCC cells. LoVo and SW480 cells were challenged with the indicated concentrations of SSa for 30 h (for sub-G1 fraction analysis) or 24 h (for Hoechst 33342 staining or annexin V staining). H1299 and H358 cells were also challenged with the indicated concentrations of SSa for 24 h for Hoechst 33342 staining. Each column represents the mean ± standard error of three independent experiments. $^{*}P < 0.05$ and $^{**}P < 0.01$ compared with DMSO control. The Mann-Whitney *U* test was used for statistical analysis.

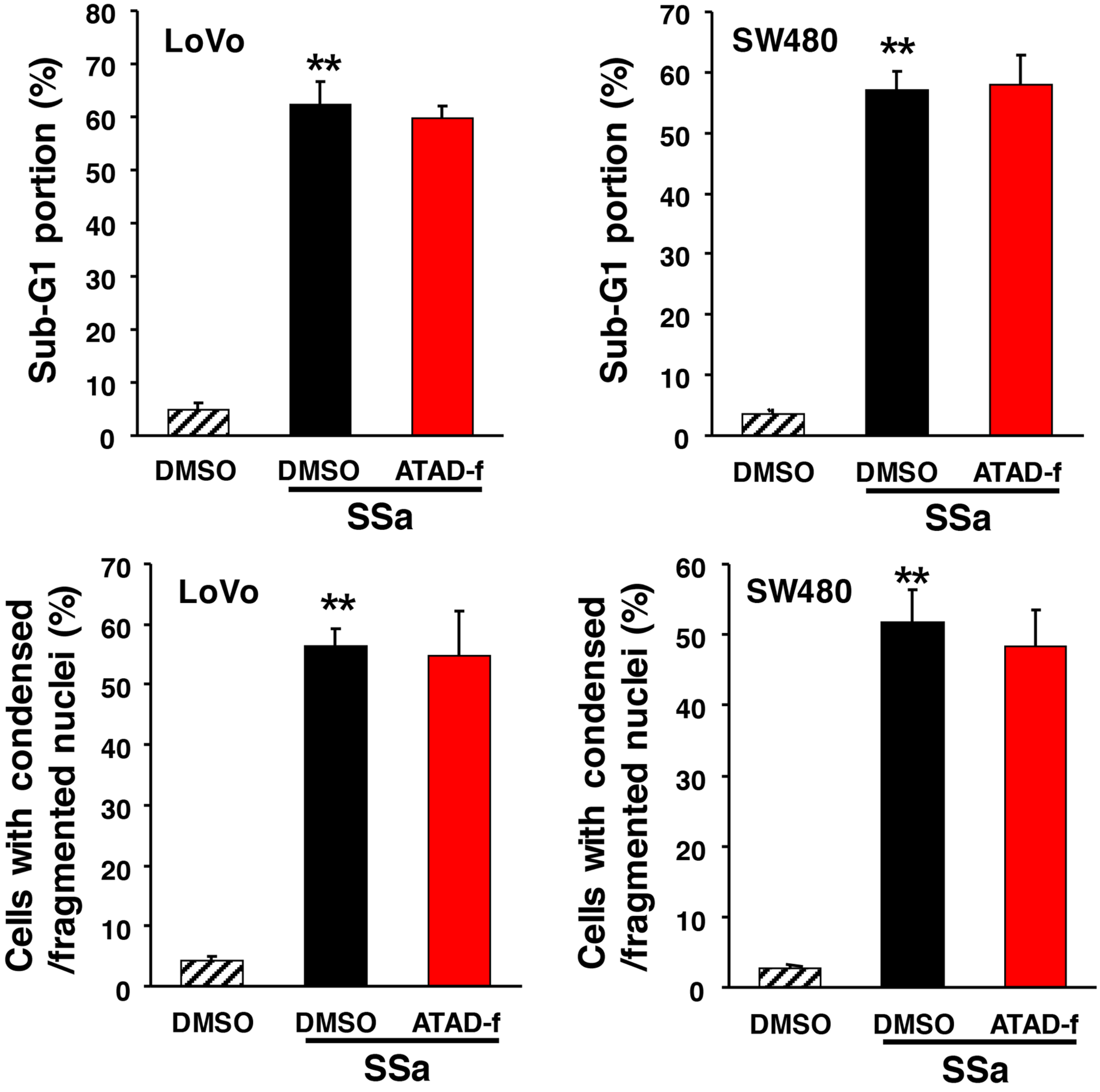

Supplementary Figure 6: Effect of caspase-12 inhibitor in SSa-induced apoptosis. LoVo and SW480 cells were exposed to 20 µM SSa for 30 h (for sub-G1 fraction analysis) or 24 h (for Hoechst 33342 staining) in the presence or absence of caspase-12 inhibitor, z-ATAD-fmk (ATAD-f; 10 µM). Each column represents the mean ± standard error for three experiments. $^{**}P < 0.01$ compared with untreated controls. ANOVA and Tukey's test were used for statistical analysis.

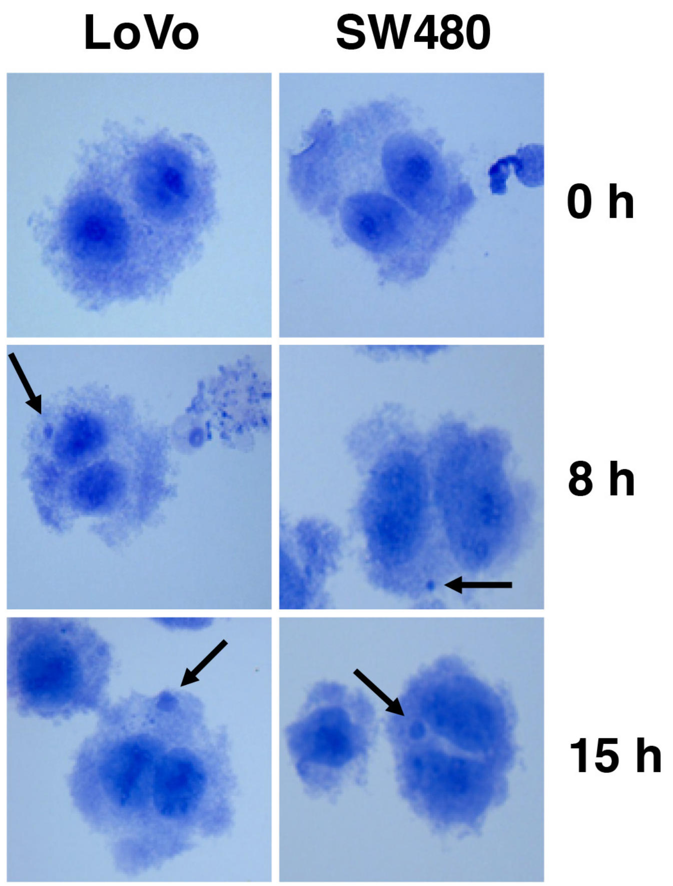

Supplementary Figure 7: Representative images of two colon cancer cells scored using the CBMN assay. Normal binucleated cells and binucleated cells with micronuclei are shown. Arrows indicate micronuclei.

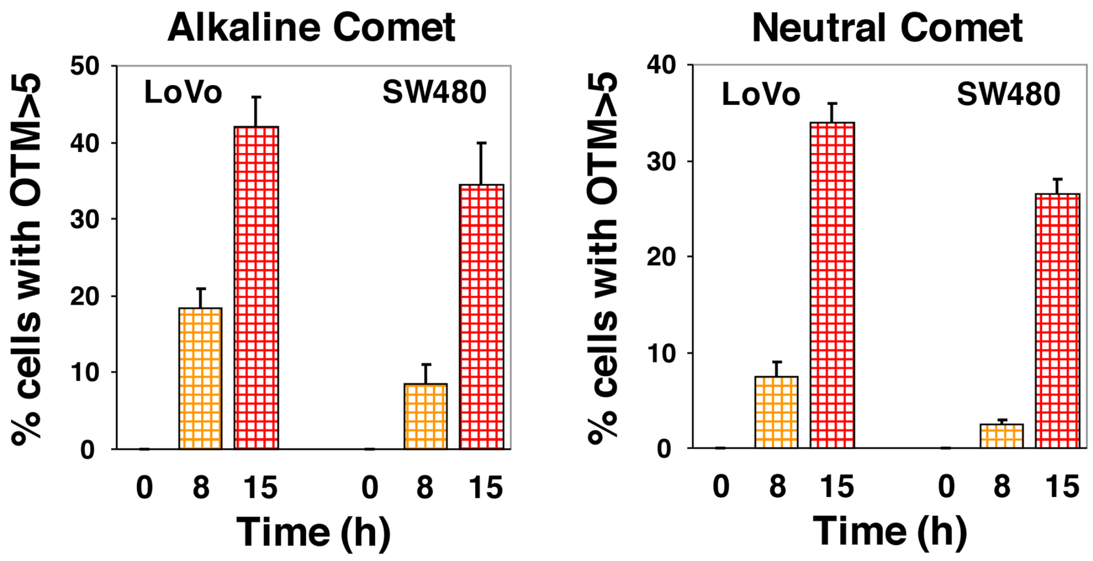

Supplementary Figure 8: The percentage of cells with OTM > 5. Results are the mean ± standard error from three experiments.

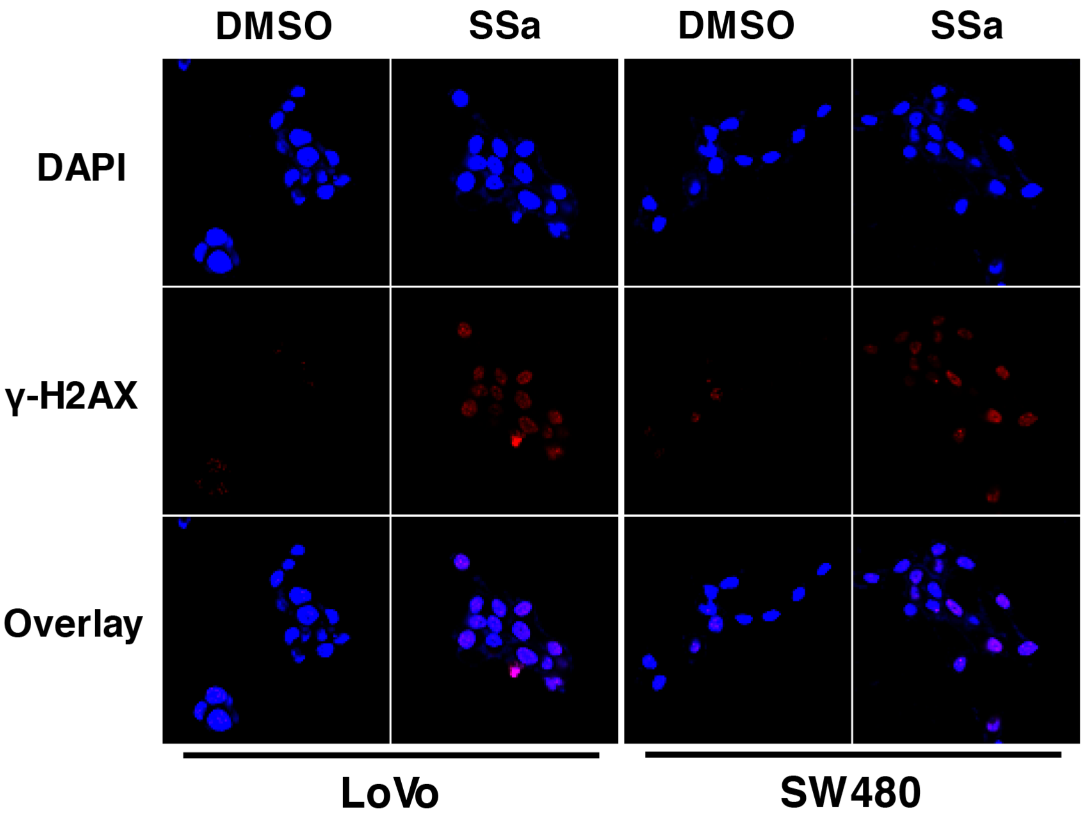

Supplementary Figure 9: Representative images of γ-H2AX and DAPI overlays.

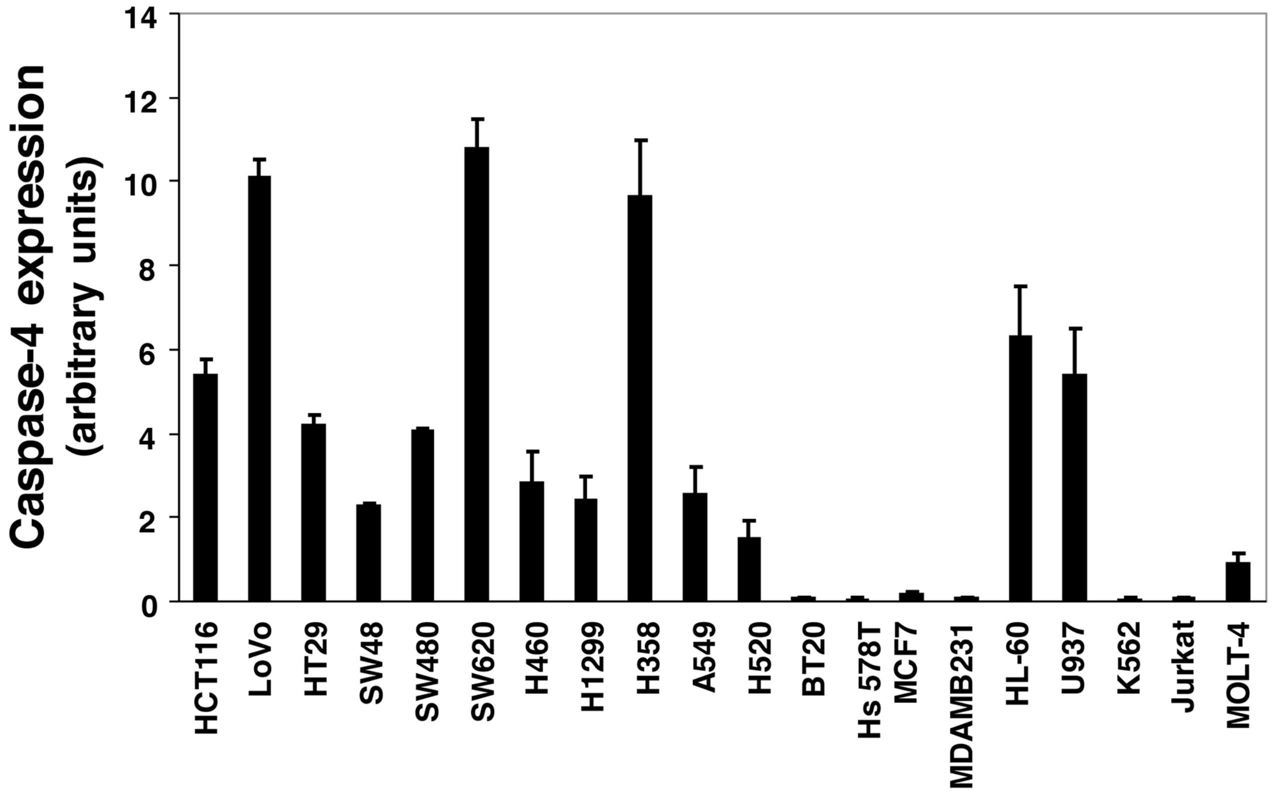

Supplementary Figure 10: Pro-caspase-4 expression in cancer cell lines derived from different tumor types. Protein levels were assessed by western blot. Data shown represent the mean ± standard error of two independent experiments.

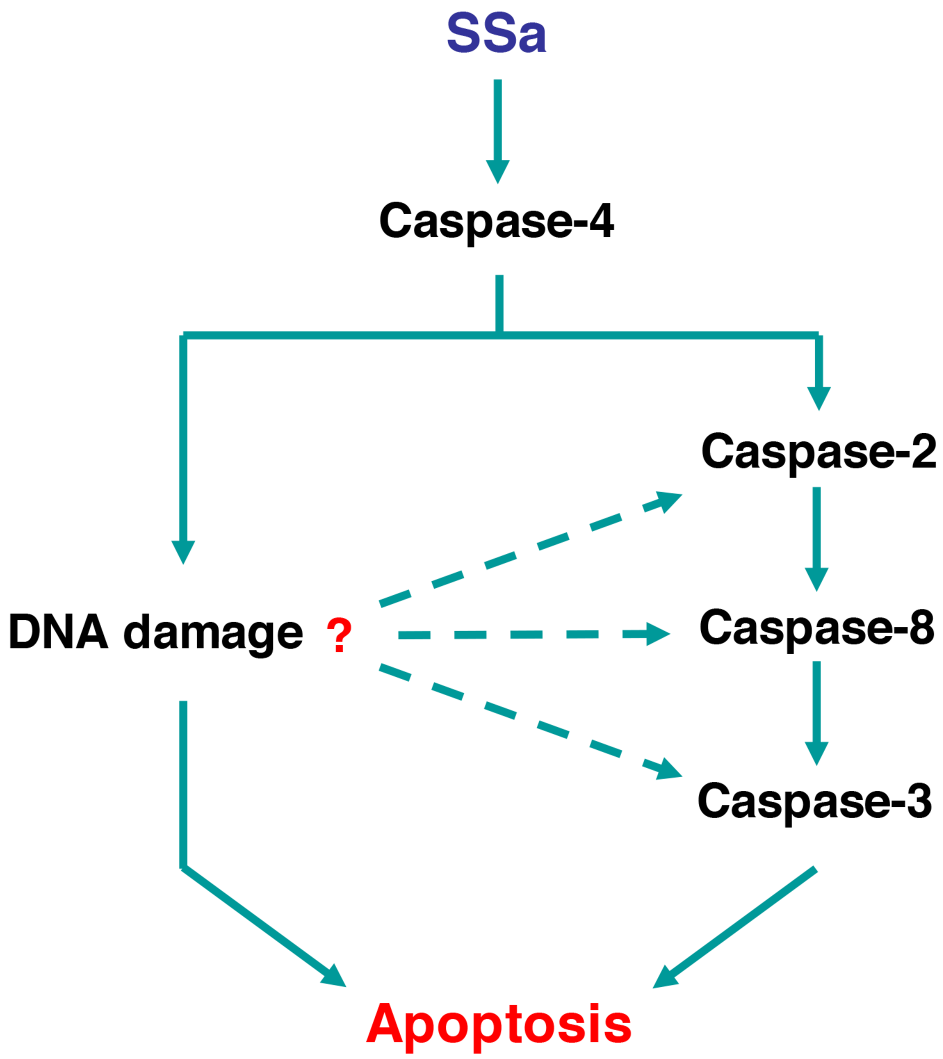

Supplementary Figure 11: Proposed model by which SSa exerts its pro-apoptotic action through caspase-4 activation followed by DNA damage and/or sequential activation of caspase-2, -8, and -3. The question mark indicates hypothetical effects of DNA damages on the activation of caspase-2, -8, and -3 that have yet to be verified.
